# Pan‐cancer analyses reveal that increased Hedgehog activity correlates with tumor immunosuppression and resistance to immune checkpoint inhibitors

**DOI:** 10.1002/cam4.4456

**Published:** 2021-11-28

**Authors:** Junjie Jiang, Yongfeng Ding, Yanyan Chen, Jun Lu, Yiran Chen, Guanghao Wu, Nong Xu, Haiyong Wang, Lisong Teng

**Affiliations:** ^1^ Department of Surgical Oncology The First Affiliated Hospital Zhejiang University School of Medicine Hangzhou China; ^2^ Department of Medical Oncology The First Affiliated Hospital Zhejiang University School of Medicine Hangzhou China; ^3^ School of Medicine Hangzhou Normal University Hangzhou China

**Keywords:** biomarker, clinical benefit, Hedgehog signaling, immune checkpoint inhibitors, immunosuppression

## Abstract

**Background:**

Immune checkpoint inhibitors (ICIs) have shown numerous clinical benefits in multiple cancer types, but good predictive biomarkers are severely lacking. Although increasing evidence has linked Hedgehog (Hh) signaling pathway with tumor development, a systematic investigation for its potential as a biomarker remains elusive.

**Methods:**

We collected and analyzed the transcriptional data and clinical outcomes of diverse cancers from the Cancer Genome Atlas and four published ICI datasets. Hh activity was estimated by conducting a single‐sample gene‐set enrichment analysis (ssGSEA) for the Hh‐related genes and calculating the ssGSEA score in each tumor sample.

**Results:**

Our findings suggest that tumors with high Hh activity displayed multiple immunosuppressive characteristics, including lack of anti‐tumor response pathways, downregulation of immune effectors, enrichment of immunosuppressive cells and chemokines, and activation of immunosuppressive signaling. Notably, patients in the non‐response group had enriched Hh activity and showed worse overall survival (OS; pooled HR = 1.50, 95% CI = 1.02–2.21, *p* = 0.039). In the subgroup of high programmed cell death ligand 1 (PD‐L1) expression, patients who harbored high Hh activity displayed a dramatically lower response rate to ICIs and a strikingly worse OS (pooled HR = 2.89, 95% CI = 1.53–5.49, *p* < 0.001).

**Conclusion:**

Increased Hh activity correlates with tumor immunosuppression across diverse cancers. Hh activity is not only a predictive biomarker for resistance to ICIs but can also better predict clinical outcomes in combination with PD‐L1 expression.

## Graphical abstract

This study highlights that increased Hedgehog (Hh) activity correlated with multiple immunosuppressive characteristics in the tumor microenvironment of diverse cancers. Moreover, Hh activity was a predictive biomarker for resistance to immune checkpoint inhibitor therapy in patients with metastatic cancer. Furthermore, combination with programmed cell death ligand 1 expression was able to better predict clinical outcomes than a single biomarker.

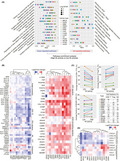

## 1 INTRODUCTION

In the past decades, the application of immune checkpoint inhibitors (ICIs) has dramatically improved the prognosis of patients with advanced or metastatic cancer, especially in melanoma^1^, ^2^ and lung cancer^3^, ^4^ However, considerable clinical heterogeneity across different populations has restricted the broad application of ICIs in various cancers, hence, promoting the development of effective biomarkers is vital to enhance tumor immunotherapy.^5^ Several well‐known biomarkers such as programmed cell death ligand 1 (PD‐L1) and tumor mutation burden (TMB) have been developed and validated in various studies.^6^, ^7^, ^8^, ^9^, ^10^ Nevertheless, several patients still receive limited or no clinical benefit from ICI therapy.^11^, ^12^ This is mainly because the single‐biomarker strategy is not accurate enough to pinpoint patients who could benefit from such treatment.^13^ Recently, growing evidence reveals that PD‐L1 expression status or TMB alone is an unstable metric that could potentially cause debatable repercussions to population classification in different cancers.^14^, ^15^, ^16^ Therefore, it is urgent to develop a novel predictive biomarker and strategy to identify the population of patients that can benefit from ICI therapy.

Hedgehog (Hh) signaling plays a critical role in several development processes, including cell proliferation, differentiation, pattern formation, and vascularization, all of which are often disrupted and uncontrollable in tumor cells.^17^ Recent studies have linked Hh signaling with tumor immunosuppression, including polarization of tumor‐associated macrophages,^18^ upregulation of PD‐L1 expression,^19^ and suppression of CD8^+^ T cells.^20^ Interestingly, a clinical retrospective study found that tumor regression induced by Hh signaling inhibition was accompanied by recruitment of cytotoxic T cells into the tumor microenvironment (TME) of a basal cell carcinoma (BCC),^21^ indicating the great potential of targeting the Hh pathway in tumor immunotherapy. However, the role of Hh signaling in the TME across diverse cancers and the potential as a biomarker for immunotherapy remains elusive.

In this study, we performed an integrated bioinformatic analysis to investigate the role of Hh signaling in the TME across 14 cancer types from the Cancer Genome Atlas (TCGA). Importantly, we explored the potential of Hh signaling as a negative biomarker for ICI therapy in four independent cohorts. Furthermore, the prediction efficiency of PD‐L1 expression was also evaluated. Notably, we further developed a joint prediction strategy by combining Hh signaling with PD‐L1 expression to identify the population who may benefit from ICI therapy.

## 2 MATERIALS AND METHODS

### 2.1 Clinical cohorts and patient samples

**FIGURE 1:**
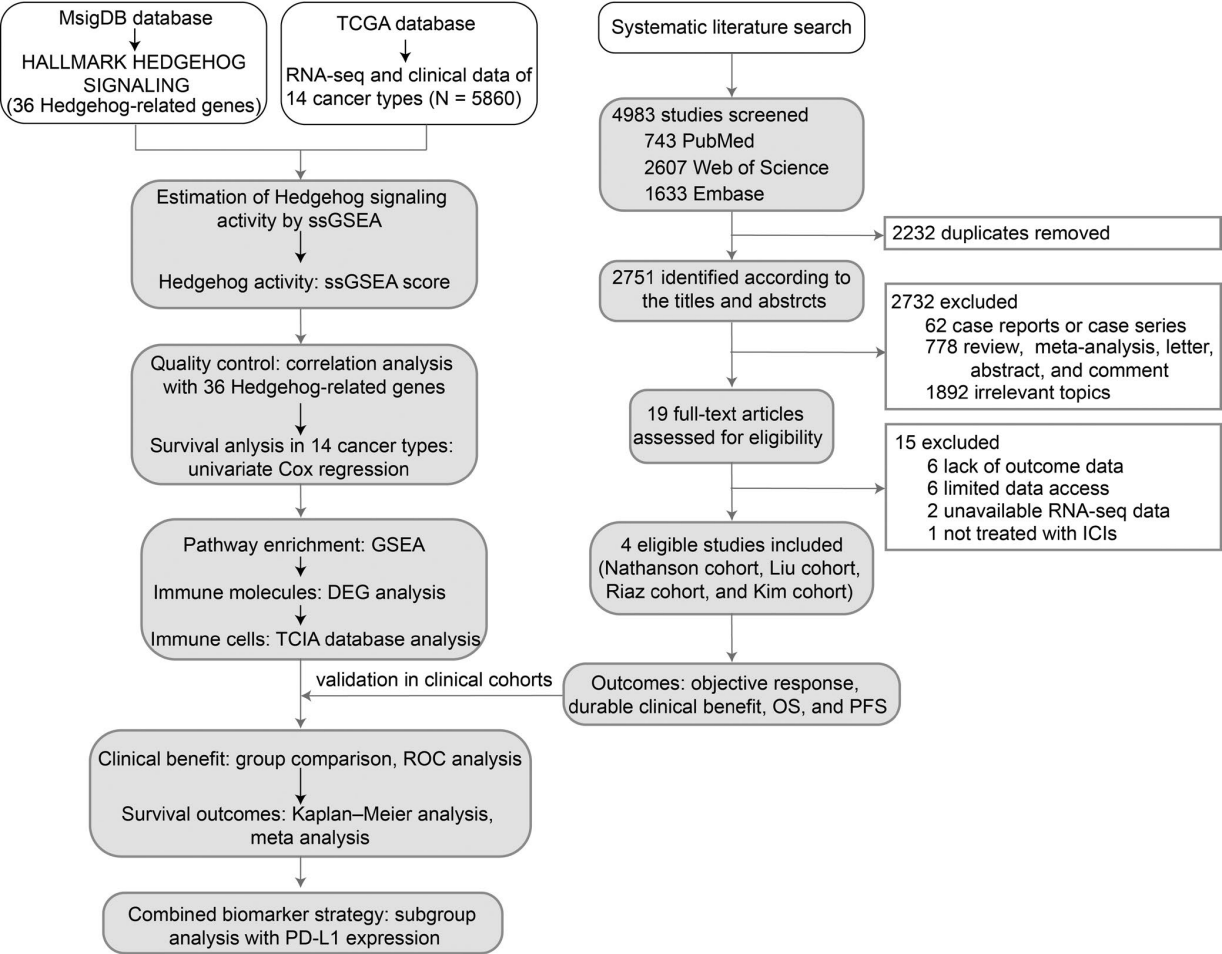
Study workflow. DEG, differentially expressed gene; ICIs, immune checkpoint inhibitors; GSEA, Gene Set Enrichment Analysis; MsigDB, Molecular Signatures Database; ROC, receiver operating characteristic; ssGSEA, single sample Gene Set Enrichment Analysis; TCGA, The Cancer Genome Atlas; TCIA, The Cancer Immunome Atlas; OS, overall survival; PFS, progression‐free survival

The study design was depicted in a workflow, as shown in Figure 1. We downloaded the RNA‐seq data and clinical information of 14 TCGA cancer types (Table 1) from the cBioPortal database (https://www.cbioportal.org/). The TGCA data analyzed in this study were released on 28 January 2016. The expression value of RNA‐seq data (RNA Seq V2 RSEM) was preprocessed by *Z*‐score standardization according to the expression distribution of each gene in all samples. A total of 5860 tumor samples were included in this study; details are shown in Table 1.

**TABLE 1:** The 14 TCGA cancer types included in this study

Cancer type	Full name	Sample size		
		Total	Low Hh activity	High Hh activity
BRCA	Breast invasive carcinoma	1100	550	550
CESC	Cervical squamous cell carcinoma and endocervical adenocarcinoma	306	153	153
GBM	Glioblastoma multiforme	166	83	83
HNSC	Head and neck squamous cell carcinoma	522	261	261
KIRC	Kidney renal clear cell carcinoma	534	267	267
KIRP	Kidney renal papillary cell carcinoma	291	146	145
LIHC	Liver hepatocellular carcinoma	373	187	186
LUAD	Lung adenocarcinoma	517	259	258
LUSC	Lung squamous cell carcinoma	501	251	250
OV	Ovarian serous cystadenocarcinoma	307	154	153
PAAD	Pancreatic adenocarcinoma	179	90	89
SKCM	Skin cutaneous melanoma	472	236	236
STAD	Stomach adenocarcinoma	415	208	207
UCEC	Uterine corpus endometrial carcinoma	177	89	88

A systematic literature search in PubMed, Web of Science, and EMBASE up to January 2021 was performed to collect published clinical cohorts associated with ICIs. The search strategy was as follows: (cancer OR carcinoma OR malignancy OR malignancies OR “malignant neoplasms” OR neoplasia OR neoplasm OR tumor) AND (PD‐1 OR PD‐L1 OR CTLA‐4 OR “immune checkpoint inhibitor” OR “immune checkpoint inhibitors” OR “ICI” OR “ICIs” OR “immune checkpoint blocker” OR “immune checkpoint blockers” OR “ICB” OR “ICBs” OR Ipilimumab OR Tremelimumab OR Nivolumab OR Pembrolizumab OR Lambrolizumab OR Atezolizumab OR Avelumab OR Durvalumab) AND (RNA‐seq OR “RNA sequencing” OR “RNA sequence” OR “Transcriptome” OR “Whole‐transcriptome sequencing” OR “Transcription sequencing” OR “Transcriptional sequencing”). The included criteria for eligibility were as follows: (1) studies associated with ICI; (2) available data on response or survival outcomes; (3) available RNA‐seq data; (4) studies published in English. Reviews, letters, comments, case reports, editorials, and abstracts were excluded. Finally, four eligible studies with clinical information and matched RNA‐seq data, including the Nathanson cohort,^22^ Liu cohort,^1^ Riaz cohort,^23^ and Kim cohort,^24^ were enrolled in this study. In the Nathanson cohort, a total of 21 patients with melanoma received ipilimumab therapy. Seven samples were collected prior to ipilimumab therapy, while 14 samples were collected after ipilimumab therapy. In the Liu cohort, 51 and 70 patients with melanoma received nivolumab and pembrolizumab, respectively. 120 samples were collected before anti‐PD1 therapy, while 1 sample was collected after anti‐PD1 therapy. In the Riaz cohort, 29 melanoma patients had previously progressed on ipilimumab therapy before receiving nivolumab therapy, while 20 patients with melanoma only received nivolumab therapy. Forty‐two tumor samples were collected before administering nivolumab, while seven tumor samples were collected during the treatment period In the Kim cohort, a total of 45 patients received nivolumab therapy and all tumor samples were obtained before initiation of study treatment. Detailed baseline characteristics of patients are shown in Table S1.

### 2.2 Estimation of Hh activity in patient samples

The HALLMARK HEDGEHOG SIGNALING geneset was obtained from the Molecular Signatures Database (MsigDB) (https://www.gsea‐msigdb.org/gsea/msigdb), containing 36 up‐regulated genes (named as Hh‐related genes) after the activation of Hh signaling.^25^ The details of the Hh‐related genes are shown in Table S2.

Single‐sample gene‐set enrichment analysis (ssGSEA) is a rank‐based method that estimates an overexpression measure for a geneset relative to other genes in the genome.^26^ It has been recognized as a powerful tool to estimate signaling activity based on transcriptome data in previous bioinformatic studies.^27^, ^28^ In this study, the ssGSEA algorithm was employed to assess the Hh activity of each sample across diverse cancers based on the gene expression level of the Hh signaling pathway. Based on the RNA‐seq data of 14 TCGA cohorts and four clinical cohorts with ICI therapy, we estimated the Hh activity by conducting ssGSEA analysis for the Hh‐related genes and calculating the ssGSEA score of each sample using the R package “GSVA”.^29^ The median ssGSEA score was adopted as the cutoff value for dividing tumor samples into groups with low and high Hh activity in each cohort.

### 2.3 Definition of clinical outcomes

In this study, best objective response, durable clinical benefit (DCB), overall survival (OS), and progressive‐free survival (PFS) were adopted as the clinical outcomes of patients treated with ICIs. The best objective response was defined using Response Evaluation Criteria in Solid Tumors (RECIST) version 1.1. DCB was defined as a composite endpoint of complete response (CR) or partial response (PR) to ICIs or stable disease (SD) with PFS more than 6 months. No durable clinical benefit was defined as progressive disease (PD) or SD with PFS less than 6 months. OS was defined as the time from the first treatment using ICIs to the date of death or censoring of data for patients without documentation of death. For patients without documentation of death, OS was censored on the last contact date with the patient. PFS was defined as the time from the first treatment of ICIs to the date of disease progression or censoring of data for patients without documentation of progression.^1^

### 2.4 Gene‐set enrichment analysis

Gene set enrichment analysis (GSEA) is a statistical method that determines whether a predefined geneset shows statistically significant, concordant differences between two biological states.^30^ GSEA is more efficient than conventional single‐gene methods to analyze coordinate pathway‐level changes in transcriptomics study.^31^ In this study, GSEA analysis for RNA‐seq data of 14 TCGA cancer types was performed using the java GSEA 3.0 Desktop Application (http://software.broadinstitute.org/gsea).^32^ We performed the GSEA analysis by comparing the gene expression profiles (GEPs) between the group with high Hh activity versus low Hh activity and conducting pathway enrichment analysis based on the KEGG and REACTOME pathways. The significant pathways enriched across all 14 cancers were considered as the common signaling associated with Hh activity and were then visualized using the R package “ggplot2”. FDR < 0.05 was considered statistically significant.

### 2.5 Evaluation of immune activity, immune cells, key signaling, and biomarkers for immunotherapy in tumors

The immune‐ and stroma‐related genesets were obtained from published literature.^33^, ^34^, ^35^, ^36^, ^37^, ^38^, ^39^ Immune‐related genesets included antigen‐processing related genes, CD8^+^ TIL markers, IFN‐γ downstream genes, NK cell markers, cytolytic effectors, and immune checkpoints. Stroma‐related genesets included the hallmark genes representing the activity of cancer‐associated fibroblast (CAF) and extracellular matrix (ECM) remodeling. Besides, we investigated the distinct expression pattern of immunostimulatory chemokines (*CXCL9*, *CXCL10*, *CXCL11*, *CCL4*, and *CCL5*) and immunosuppressive chemokines (*CXCL8*, *CXCL5*, *CXCL12*, *CCL2*, and *CCL22*) between groups with low and high Hh activity. The fold change of transcriptional expression in these genes was calculated using the software R package limma.^40^ Fold change > 1.5 and FDR < 0.05 were set as the cut‐off values. Red or blue in the heatmaps represent up‐or down‐regulation of gene expression in the group with high Hh activity, comparing to those with low Hh activity, respectively. In addition, the profile of tumor‐infiltrating immune cells fractions in 14 TCGA cancer types was downloaded from The Cancer Immunome Atlas database (TCIA) (https://tcia.at/). The immune cell types included CD8^+^ T cells, CD4^+^ T cells, regulatory T cells, macrophage M1 and M2, estimated by the quanTIseq deconvolution algorithm.^41^ CIBERSORT was a deconvolution algorithm to estimate the cell proportion of 22 immune cell types with an immune geneset by support vector regression.^42^ It has been widely employed in previous studies to evaluate tumor immune infiltrating cells in tumor samples based on transcriptome data.^43^, ^44^ We also performed the CIBERSORT algorithm to characterize the detailed types of immune cells infiltrating the tumors. Then we comprehensively compared the relative fractions of these immune cells between the groups with low and high Hh activity. Furthermore, transforming growth factor‐beta (TGF‐β) and Wnt signaling played key roles in resistance to ICI therapy as previously reported.^45^, ^46^ In this study, we performed ssGSEA to estimate the pathway activity of TGF‐β and Wnt signaling in each sample, then compared them with low and high Hh activity. The predictive biomarkers including cytolytic activity (CYT),^38^ T cell–inflamed GEP,^47^ IFN‐γ signature (IFN‐γ),^48^ and MHC class I antigen‐presenting machinery expression (APM)^49^ were estimated based on the transcriptional data and the corresponding geneset provided in the original articles.

### 2.6 Survival analysis and ROC analysis

The prognostic value of Hh signaling in 14 TCGA cancer types was evaluated using univariate Cox regression and visualized using the R package “forestplot.” Patients in the Nathanson cohort, Liu cohort, and Riaz cohort were grouped by the median cutoff of a specific variable (Hh activity, PD‐L1 expression, and TMB), then compared using Kaplan–Meier analysis to evaluate the difference in survival outcomes (OS and PFS). Meta‐analysis was performed to estimate the summary prognostic effect and the heterogeneity among the independent cohorts using the R package “meta”.^50^ Subgroup analysis was conducted to preliminarily explore the feasibility and significance of biomarker‐combination strategy between Hh signaling, PD‐L1, and TMB for predicting response to ICI therapy. The median value of PD‐L1 expression or TMB was adopted as the cutoff in the subgroup analysis. Receiver operating characteristic (ROC) analysis was conducted using the R package “pROC”^51^ to evaluate the predictive value of Hh signaling for resistance to ICI therapy.

### 2.7 Total RNA isolation and real‐time polymerase chain reaction

A total of 10 tumor samples of patients with gastric cancer (GC) were obtained from the First Affiliated Hospital of Zhejiang University. Eight samples were collected from GC patients receiving neoadjuvant immunotherapy, while two samples were collected from GC patients receiving adjuvant immunotherapy. Total RNA of tumor samples was extracted using RNeasy Mini Kit (Cat. no. 74106; Qiagen) and quantified using NanoDrop One (Cat. ND‐ONE‐W; ThermoFisher Scientific). Real‐time PCR was performed using PrimeScript™ RT Master Mix (Perfect Real Time) (Cat. #RR036A; TaKaRa) and TBGreen^®^Premix Ex Taq™II (Tli RNase H Plus) (Cat. #RR820A; TaKaRa) according to the manufacturer's instruction. Glyceraldehyde‐3‐phosphate dehydrogenase (GAPDH) was used as an endogenous control.

### 2.8 Statistical analysis

Statistical analyses were performed using SPSS software (version 21.0; IBM Corp.) and GraphPad Prism (version 6.01). Spearman's correlation was used to examine the correlation between Hh activity and expression level of the Hh‐related genes, as well as the predictive biomarkers for immunotherapy (PD‐L1 expression, CYT, GEP, IFN‐γ, APM, and TMB). The prognostic value of Hh activity in 14 TCGA cancer types was evaluated using the hazard ratio (HR) and corresponding 95% confidence interval (CI) from univariate Cox regression analysis. The fold change of gene expression between the group with high Hh activity versus low Hh activity was calculated using the R package “limma”.^40^ Fold change > 1.5 and FDR < 0.05 were set as the cut‐off values. The comparisons of immune cell fractions and WNT/TGF‐β signaling between the group with low and high Hh activity were conducted using the Mann–Whitney test. The differences in Hh activity and Hh‐related gene expression level between response and non‐response groups were also determined using the Mann–Whitney test. The predictive power of Hh activity for resistance to immunotherapy was evaluated using the area under the curve (AUC) from ROC analysis. Kaplan–Meier analysis with log‐rank test was performed to evaluate the association between Hh activity and survival outcomes of patients receiving ICI therapy. In the meta‐analysis, the value of I^2^ represented the heterogeneity level as follows: low (*I* ^2^ < 25%), moderate (*I* ^2^ = 25%–75%), or high (*I* ^2^ > 75%). A random‐effects model was applied for meta‐analysis. The publication bias was estimated using Begg's test and Egger's test. The response rate difference between the group with low and high PD‐L1 expression/TMB was compared by the *χ* ^2^ test or Fisher's exact test. All reported *P* values were two‐tailed, and *p <* 0.05 was considered statistically significant.

## 3 RESULTS

### 3.1 The estimation, correlation, and survival analysis of Hh activity in diverse cancers

**FIGURE 2:**
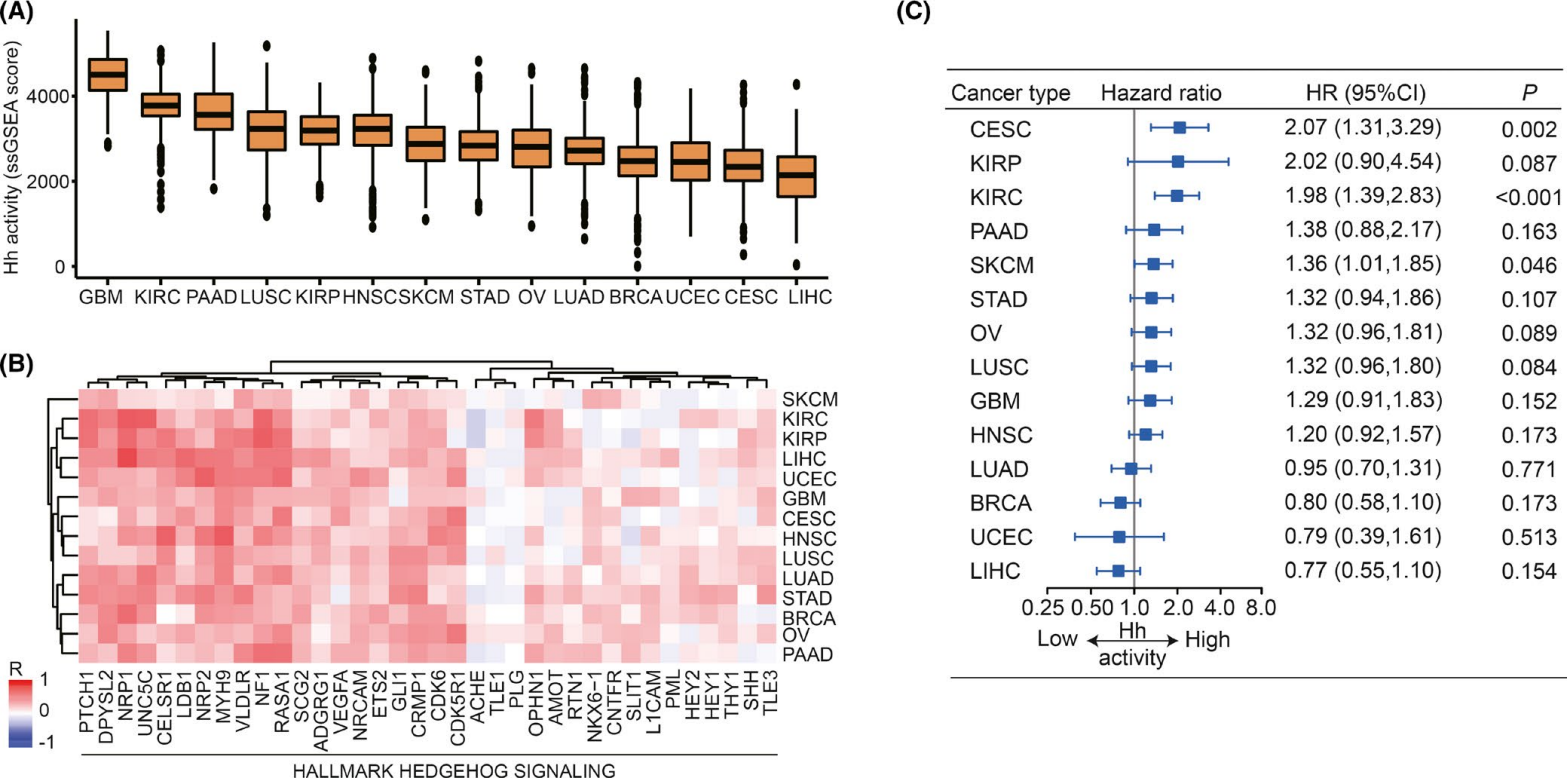
Estimation of Hedgehog activity and survival analysis in diverse cancers. (A) Boxplots of Hh activity showing variation across 14 cancer types. The Hh activity of each sample is estimated by ssGSEA analysis. A higher ssGSEA score represents the higher activity of Hh signaling. (B) Heatmap showing the Spearman's correlation between Hh activity (ssGSEA score) and the transcriptional expression level of 36 Hedgehog‐related genes across 14 cancer types. The Hedgehog‐related genes contained in the HALLMARK HEDGEHOG SIGNALING geneset were obtained from the MsigDB database. Red and blue represent the positive and negative correlation, respectively. (C) Forest plot of univariate Cox regression analysis showing the association between Hedgehog activity and overall survival across 14 cancer types. The median value of Hh activity was adopted as the threshold for the groups with low and high Hh activity. The hazard ratios are presented and the horizontal lines indicate the 95% confidence intervals. Hh, Hedgehog; HR, hazard ratio; CI, confidence interval; ssGSEA, single sample Gene Set Enrichment Analysis

Initially, upon acquiring 36 Hh‐related genes from MsigDB database, we performed ssGSEA to estimate the Hh activity across 14 TCGA cancer types, including BRCA, CESC, GBM, HNSC, KIRC, KIRP, LIHC, LUAD, LUSC, OV, PAAD, SKCM, STAD, and UCEC (Table 1). There was a broad variation of Hh activity in diverse cancers (Figure 2A). The median Hh activity was relatively high in GBM, KIRC, and PAAD, while low in UCEC, CESC, and LIHC. We then performed correlation analysis between Hh activity and the transcriptional expression of 36 Hh‐related genes; 91.7% (33/36) Hh‐related genes showed positive correlation with Hh activity in 14 TCGA cancer types (Figure 2B; Table S3). Subsequently, we performed univariate Cox regression to examine the prognostic impact of Hh activity in 14 TCGA cancer types (Figure 2C), which revealed that high Hh activity had a significantly increased risk of death in CESC (HR = 2.07, 95% CI = 1.31–3.29, *p* = 0.002), KIRC (HR = 1.98, 95% CI = 1.39–2.83, *p *< 0.001) and SKCM (HR = 1.36, 95% CI = 1.01–1.85, *p* = 0.046). Besides, patients with high Hh activity tended to have a poor survival outcome in KIRP (HR = 2.02, 95% CI = 0.90–4.54, *p* = 0.087), STAD (HR = 1.32, 95% CI = 0.94–1.86, *p* = 0.107), OV (HR = 1.32, 95% CI = 0.96–1.81, *p* = 0.089) and LUSC (HR = 1.32, 95% CI = 0.96–1.80, *p* = 0.084), although it was not significant (Figure 2C). In addition, prognostic value of Hh activity was absent in other cancer types with low median Hh activity, such as LUAD (HR = 0.95, 95% CI = 0.70–1.31, *p* = 0.771), BRCA (HR = 0.80, 95% CI = 0.58–1.10, *p* = 0.173), UCEC (HR = 0.79, 95% CI = 0.39–1.61, *p* = 0.513), and LIHC (HR = 0.77, 95% CI = 0.55–1.10, *p* = 0.154). When adjusted by age, the association between Hh activity and OS in CESC (HR = 2.05, 95% CI = 1.29–3.26, *p* = 0.002) and KIRC (HR = 2.00, 95% CI = 1.37–2.91, *p *< 0.001) were still significant, whereas not significant in other cancer types (Figure S1).

### 3.2 The role of Hh signaling in the TME of diverse cancers

**FIGURE 3:**
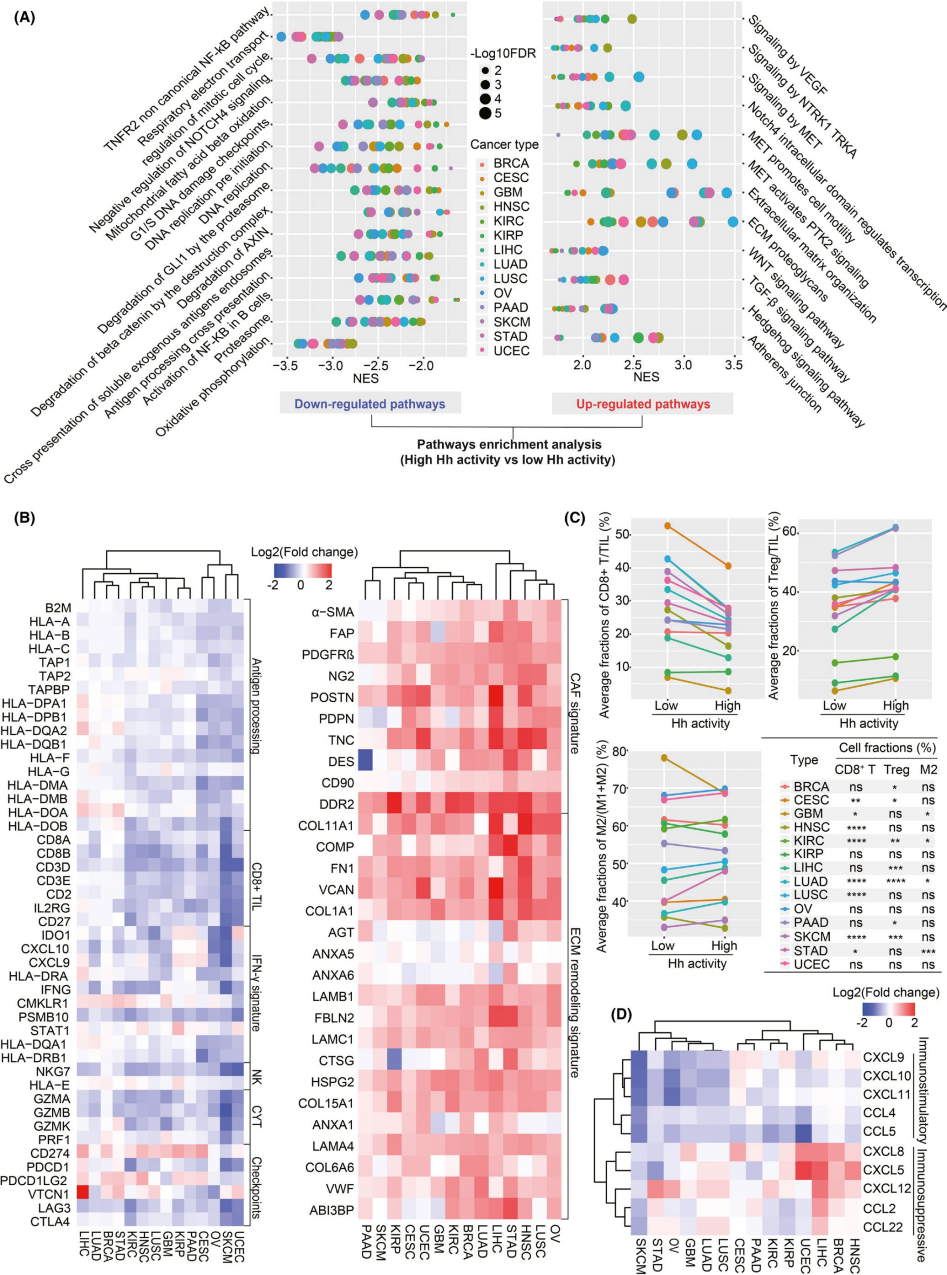
Association between Hedgehog activity and the tumor microenvironment in diverse cancers. (A) Representative pathways enriched in the group with low Hh activity (left panel) and high Hh activity (right panel) across 14 cancer types. All tumor samples in each cancer type were divided into two groups according to the median value of Hh activity. Based on the KEGG and REACTOME pathway datasets, GSEA analysis was performed to identify the significant pathways enriched in the group with low and high Hh activity. FDR < 0.05 was considered statistically significant. The horizontal axis shows the normalized enrichment score and the vertical axis shows the representative pathways enriched in the group with low and high Hh activity. Different cancer types are labeled with different colors. KEGG, Kyoto Encyclopedia of Genes and Genomes; NES, normalized enrichment score; FDR, false discovery rate. (B) Heatmaps showing fold changes of immune‐related (upper panel) or stroma‐related (lower panel) gene expression in the groups with high Hh activity versus low Hh activity. Rows show gene symbols and columns show cancer types. Annotation bar indicates the fold change of gene expression in the groups with high Hh activity versus low Hh activity. Immune‐related genes include antigen‐processing related genes, CD8^+^ TIL markers, IFN‐γ downstream genes, NK cell markers, cytolytic effectors, and immune checkpoints. Stroma‐related genes include CAF‐signature genes and ECM remodeling‐related genes. Red and blue represent up‐regulation and down‐regulation of the gene expression in the groups with high Hh activity comparing to those with low Hh activity, respectively. Fold change > 1.5 and FDR < 0.05 were set as the cut‐off values. CAF, cancer‐associated fibroblast; ECM, extracellular matrix; NK, natural killer cells; CYT, cytolytic activity. (C) Comparisons of the relative fractions of CD8^+^ T/TIL, Treg /TIL, and M2/(M1+M2) between the groups with low and high Hh activity across 14 TCGA cancer types. The horizontal axis shows the groups with low and high Hh activity. The vertical axis shows the median value of average immune cell fractions in each cancer type. Different cancer types are labeled with different colors. The table shows the statistical differences of relative immune cell fractions between the groups with low and high Hh activity. TIL, tumor‐infiltrating lymphocytes; Treg, regulatory T cells; M1, classical M1 macrophage; M2, alternative M2 macrophage. *p < 0.05, **p < 0.01, ***p < 0.001, ****p < 0.0001; ns, not significant. (D) Heatmap showing the fold changes of the chemokine expression at the transcriptional level in the groups with high Hh activity versus low Hh activity. Rows show gene symbols, including immunostimulatory chemokines (*CXCL9*, *CXCL10*, *CXCL11*, *CCL4*, and *CCL5*) and immunosuppressive chemokines (*CXCL8*, *CXCL5*, *CXCL12*, *CCL2*, and *CCL22*). Columns show cancer types. Annotation bar indicates the fold change of gene expression in the groups with high Hh activity versus low Hh activity

To understand the role of Hh signaling in the TME across diverse cancers, we performed GSEA analysis based on the GEPs of 14 TCGA cancer types and identified the KEGG/REACTOME pathways significantly enriched in the tumors with low and high Hh activity, respectively. Most of the enriched pathways in low Hh activity tumors were associated with cell cycle and anti‐tumor immune response (Figure 3A; Table S4). The immune response pathway included TNFR2 non‐canonical nuclear factor‐kappaB (NF‐κB) pathway, cross‐presentation of soluble exogenous antigens endosomes, antigen processing cross‐presentation, and activation of NF‐κB in B cells, suggesting the presence of an active immune microenvironment in low Hh activity tumors. In contrast, tumors with high Hh activity showed active signaling of the receptor tyrosine kinase MET and ECM (Figure 3A; Table S4). The ECM‐related pathways included vascular endothelial growth factor (VEGF) signaling, ECM organization, ECM proteoglycans, and TGF‐β signaling, indicating that high Hh activity was associated with TGF‐β‐associated ECM remodeling, a potential promoter for tumor immunosuppression.^34^ Subsequently, we investigated the differentially expressed genes involved in immune‐related and stroma‐related signatures by comparing tumors with high to low Hh activity. We found that, especially in UCEC, SKCM, OV, and CESC, tumors with high Hh activity had a relative lack of immune effectors but showed an abundance in immune checkpoints, the former including antigen processing genes, CD8^+^ TIL markers, IFN‐γ related genes, NK cell markers, and cytolytic effectors, and the latter including CD274 (also named PD‐L1), PD‐L2, and VTCN1 (Figure 3B). As expected, compared with tumors with low Hh activity, tumors with high Hh activity showed dramatically active signatures of CAFs and ECM remodeling in 14 cancer types, such as the upregulated expression of COL11A1, DDR2, COMP, FN1, VCAN, and COL1A1, most of which correlated with tumor immunosuppression (Figure 3B).

Furthermore, based on the TCIA data, we found significantly different fractions of immune cells between tumors with low and high Hh activity. Compared with tumors with low Hh activity, tumors with high Hh activity showed significantly lower fractions of CD8^+^ T cells, the major effector for anti‐tumor immune response, especially in CESC (*p *< 0.01), GBM (*p *< 0.05), HNSC (*p *< 0.0001), KIRC (*p *< 0.0001), LUAD (*p *< 0.0001), LUSC (*p *< 0.0001), SKCM (*p *< 0.0001), and STAD (*p *< 0.05) (Figure 3C). Interestingly, regulatory T cells were significantly enriched in tumors with high Hh activity, especially in BRCA (*p *< 0.05), CESC (*p *< 0.05), KIRC (*p *< 0.01), LIHC (*p *< 0.001), LUAD (*p *< 0.0001), PAAD (*p *< 0.05), and SKCM (*p *< 0.001) (Figure 3C). Also, the association between Hh activity and macrophage 2 (M2) cells showed an inconsistent trend across 14 cancer types. Tumors with high Hh activity harbored more abundance of M2 cells in KIRC (*p *< 0.05), LUAD (*p *< 0.05), and STAD (*p *< 0.001) but less in GBM (*p *< 0.05). We further conducted CIBERSORT to validate the different abundance of immune cells between the groups with low and high Hh activity. As shown in Figure S2, the high Hh activity group contained significantly decreased CD8^+^ T cells and increased Treg cells compared with the group with low Hh activity in most cancer types. Besides, the memory CD4^+^ T cells also showed an unbalanced distribution; the resting type was enriched in the group with high Hh activity, while the activated type was enriched in the group with low Hh activity. Furthermore, the group with low Hh activity was more abundant in activated NK cells and macrophage M1 cells in several cancer types, such as LUAD, LUSC, and SKCM. Moreover, we investigated the expression of several chemokines, which serve as key regulators for the chemotaxis of immune cells in the TME. We observed that immunostimulatory chemokines such as CXCL9, CXCL10, CXCL11, CCL4, and CCL5 were consistently down‐regulated for the tumors with high Hh activity across the 14 cancer types. However, immunosuppressive chemokines such as CXCL8, CXCL5, CXCL12, CCL2, and CCL22 were generally upregulated across the 13 cancer types (Figure 3D). Besides, we explored the association between Hh activity and the known pathways contributing to immunotherapy resistance, including Wnt signaling^46^ and TGF‐β signaling.^45^ Interestingly, the ssGSEA scores of Wnt signaling and TGF‐β signaling were both significantly higher in tumors with high Hh activity than low Hh activity across at least 13 cancer types (Figure S3).

Together, these findings support that increased Hh activity correlated with multiple immunosuppressive characteristics across diverse cancers and a decreased likelihood for clinical response to ICIs (Figure S4).

### 3.3 Predicting clinical outcomes of patients treated with ICIs by Hh activity, PD‐L1 expression, or TMB alone

**FIGURE 4:**
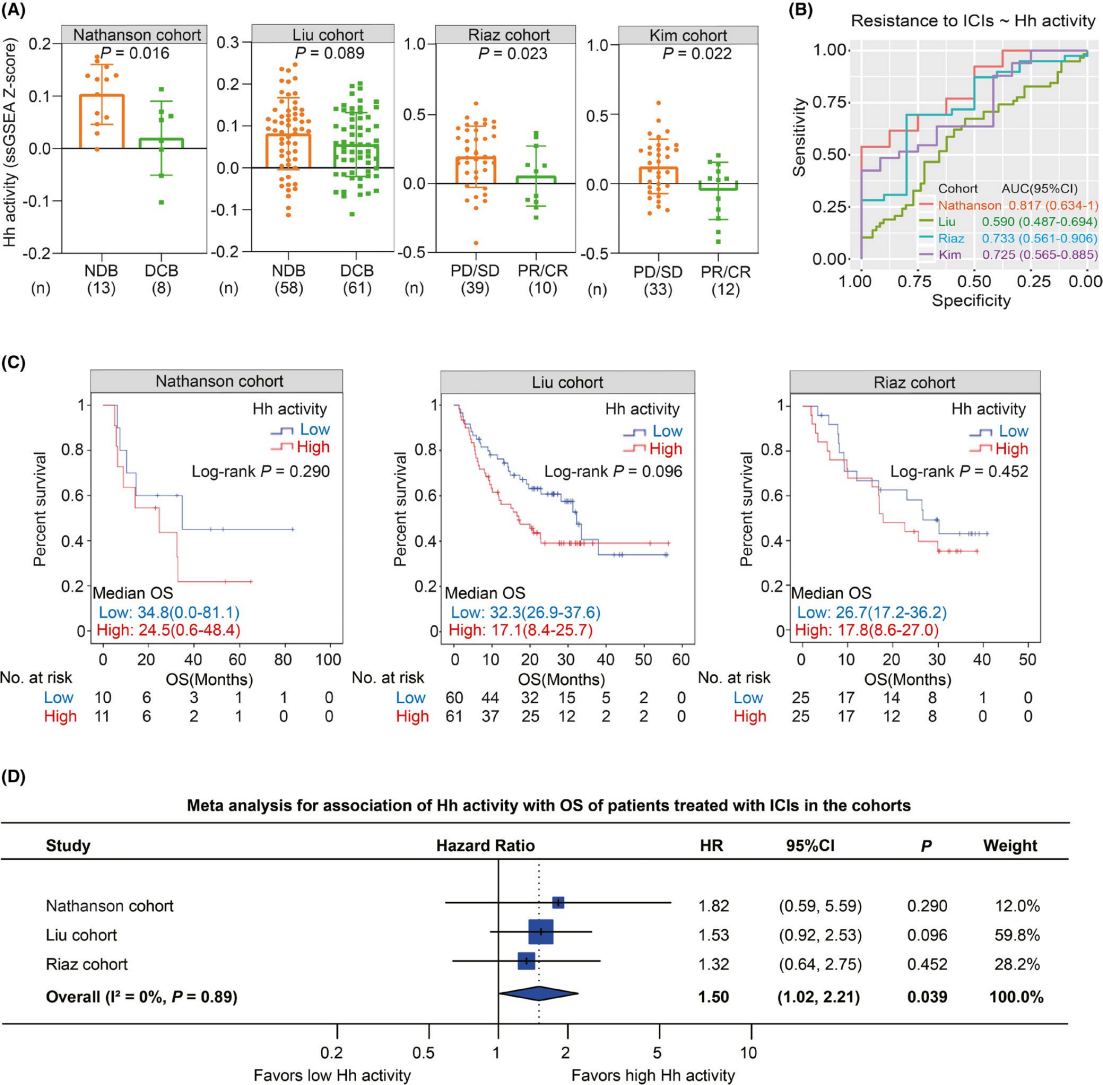
Predicting clinical outcomes of patients treated with ICIs by Hedgehog activity alone. (A) Histograms showing the comparison of Hh activity between the groups with NDB and DCB (in the Nathanson cohort and the Liu cohort) or PD/SD and PR/CR (in the Riaz cohort and the Kim cohort). (B) ROC curves for predicting resistance to ICI therapy by Hh activity in the Nathanson cohort (orange), Liu cohort (green), Riaz cohort (blue), and Kim cohort (purple). The status of NDB (in the Nathanson cohort and the Liu cohort) or PD/SD (in the Riaz cohort and the Kim cohort) was defined as resistance to ICI therapy for ROC analysis. The values of AUC with corresponding 95% CI are presented in each cohort. AUC, area under the curve; ROC, receiver operating characteristic. (C) Kaplan–Meier curves showing the association between Hh activity and OS in the Nathanson cohort (left panel), Liu cohort (middle panel), and Riaz cohort (right panel). The median value of Hh activity is adopted as the threshold for grouping patients in each cohort. The statistical significance is determined using a log‐rank test. (D) Forest plot showing the meta‐analysis for the prognostic value of Hh activity in the Nathanson cohort, Liu cohort, and Riaz cohort. The value of *I* ^2^ represented the heterogeneity level as follows: low (*I* ^2^ < 25%), moderate (*I* ^2^ = 25–75%), or high (*I* ^2^ > 75%). A random‐effects model was applied for this meta‐analysis. The hazard ratios in each cohort are presented and the horizontal lines indicate the 95% confidence intervals. Melanoma patients in the Nathanson cohort received anti‐CTLA4 therapy, whereas melanoma patients in the Liu cohort and Riaz cohort received anti‐PD‐1 therapy. Patients with gastric cancer in the Kim cohort received anti‐PD‐1 therapy. CR, complete response; DCB, durable clinical benefit; NDB, no durable clinical benefit; PD, progressive disease; PR, partial response; ROC, receiver operating characteristic; SD, stable disease

Through literature search and screening, we collected four clinical cohorts to explore the prediction of clinical outcomes in patients treated with ICIs using Hh activity, PD‐L1 expression, or TMB alone. We found that, compared to the response group, the non‐response group showed higher Hh activity in the four independent cohorts (Nathanson cohort: *p* = 0.016; Liu cohort: *p* = 0.089; Riaz cohort: *p* = 0.023; Kim cohort: *p* = 0.022; Figure 4A). As expected, the group with high PD‐L1 expression had more patients acquiring clinical benefit than the group with low PD‐L1 expression in four cohorts (Nathanson cohort: *p* = 0.01; Liu cohort: *p* = 0.41; Riaz cohort: *p* = 0.04; Kim cohort: *p* = 0.02; Figure S5A). Nevertheless, we did not observe a significant difference in clinical benefit between the subgroups with low and high TMB (Nathanson cohort: *p* = 0.10; Liu cohort: *p* = 0.12; Figure S6A). Furthermore, ROC analysis revealed that Hh activity was capable of predicting resistance to ICIs (Nathanson cohort: AUC = 0.817; Liu cohort: AUC = 0.590; Riaz cohort: AUC = 0.733; Kim cohort: AUC = 0.725; Figure 4B). Subsequently, we evaluated the prognostic value of Hh activity, PD‐L1 expression, and TMB in the patients treated with ICIs. The prognostic value of Hh activity was found to be not significant (Nathanson cohort: HR = 1.82, 95% CI = 0.59–5.59, *p* = 0.290; Liu cohort: HR = 1.53, 95% CI = 0.92–2.53, *p* = 0.096; Riaz cohort: HR = 1.32, 95% CI = 0.64–2.75, *p* = 0.452; Figure 4C,D). On the other hand, meta‐analysis identified Hh activity as a significant risk factor for OS in patients treated with ICIs (HR = 1.50; 95% CI = 1.02–2.21; Figure 4D). In the bias analysis, both Begg's test (*p* = 0.60) and Egger's test (*p* = 0.79) suggested that the publication bias was not significant. Nevertheless, in the sensitivity analysis, omitting one of the included cohorts led to the insignificant association between Hh activity and OS of patients receiving ICI therapy (Figure S7). To validate the association between Hh activity and clinical outcomes in patients treated with ICIs, we firstly collected a total of 10 patient samples who received neoadjuvant/adjuvant immunotherapy from our institution, then detected gene transcriptional expression levels of *GLI1* and *SHH* (two key genes in Hh signaling pathway^52^, ^53^) using real‐time polymerase chain reaction (RT‐PCR), and explored the clinical correlation and prognostic value of *GLI1* and *SHH*. As shown in Figure S8A, the response group (PR/CR) tended to harbor lower expression of *GLI1* and *SHH* than the non‐response group (PD/SD), although it was not statistically significant (*p* = 0.071). Survival analysis revealed that patients with low expression of *GLI1* achieve a better OS than those with high expression of *GLI1* (Median OS: 29.0 vs. 20.0, *p* = 0.042; Figure S8B). A similar trend was also observed in *SHH* (*p* = 0.133; Figure S8B). Therefore, these findings preliminarily validated that low Hh activity correlated with a high response rate and better survival outcomes in patients receiving ICI therapy. Besides, patients with high PD‐L1 expression were more likely to achieve a better OS in the Nathanson cohort (HR = 0.25, 95% CI = 0.08–0.83, *p* = 0.015; Figure S5B,C) and Riaz cohort (HR = 0.50, 95% CI = 0.24–1.05, *p* = 0.060; Figure S5B,C), as well as in the meta cohort (HR = 0.56, 95% CI = 0.30–1.03; Figure S5C), although there was low heterogeneity between these cohorts (I^2^ = 49%, *p* = 0.14; Figure S5C). However, the association between PD‐L1 expression and OS was not significant in the Liu cohort (HR = 0.84, 95% CI = 0.51–1.39, *p* = 0.502; Figure S5B,C). In addition, TMB served as a significantly protective factor of OS in the Liu cohort (HR = 0.44, *p* = 0.002; Figure S6B) in the contrast to the Nathanson cohort (HR = 0.39, *p* = 0.090; Figure S6B). Moreover, we also evaluated PFS prediction with Hh activity, PD‐L1 expression, and TMB in the Liu cohort. As a result, Hh activity was identified as a significant risk factor for PFS in the Liu cohort (HR = 1.75, *p* = 0.011; Figure S9A). However, the association of PD‐L1 expression with PFS was not significant (HR = 0.93, *p* = 0.728; Figure S9B). Besides, compared to the patients with low TMB, the patients with high TMB tended to achieve a better PFS in the Liu cohort (HR = 0.67, *p* = 0.060; Figure S9C). Taken together, these findings indicate that Hh activity, PD‐L1 expression, and TMB were potential biomarkers for predicting clinical outcomes of patients treated with ICIs; however, the single‐biomarker strategy showed unstable prediction efficiency in different populations and undeniable heterogeneity between the independent cohorts.

### 3.4 Predicting clinical outcomes of patients treated with ICIs by the combination of Hh activity with PD‐L1 expression or TMB

**FIGURE 5:**
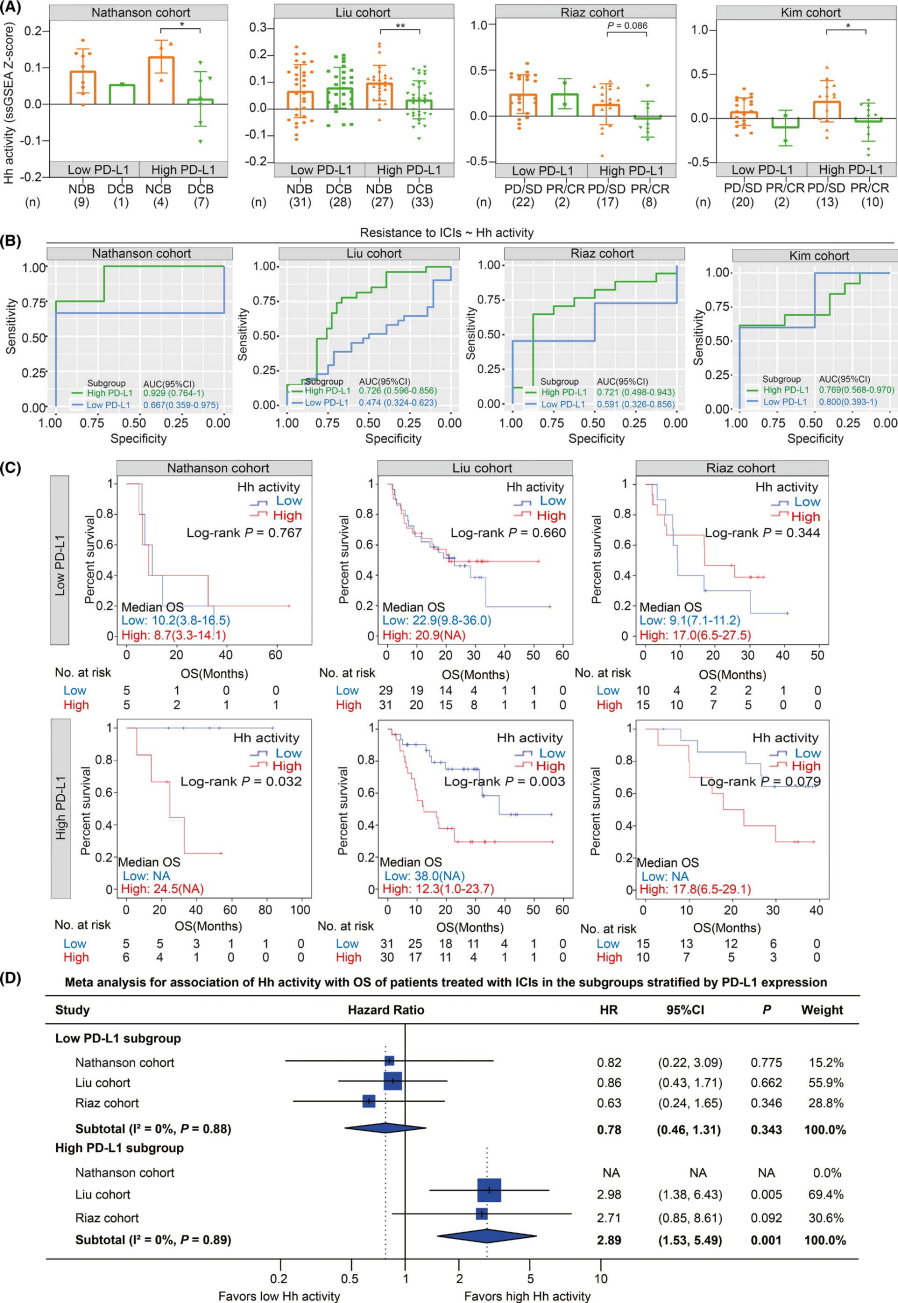
Predicting clinical outcomes of patients treated with ICIs by the combination between Hedgehog activity and PD‐L1 expression. (A) Histograms showing the association between Hh activity and response to ICI therapy in the subgroups stratified by PD‐L1 expression in the Nathanson cohort, Liu cohort, Riaz cohort, and Kim cohort. All tumor samples in each cohort were divided into two subgroups according to the median value of the PD‐L1 expression. The comparison of Hh activity was conducted between the response group (DCB or PR/CR group) and the non‐response group (NDB or SD/PD group) within the subgroups with low and high PD‐L1 expression. (B) ROC curves for predicting resistance to ICI therapy by Hh activity in the subgroups stratified by PD‐L1 expression in the Nathanson cohort, Liu cohort, Riaz cohort, and Kim cohort. (C) Kaplan–Meier curves showing the association between Hh activity and OS in the subgroups stratified by PD‐L1 expression in the Nathanson cohort (left panel), Liu cohort (middle panel), and Riaz cohort (right panel). The median value of Hh activity is adopted as the threshold for grouping patients in each cohort. The statistical significance is determined using a log‐rank test. (D) Forest plot showing meta‐analysis for the prognostic value of Hh activity in the subgroups stratified by PD‐L1 expression in the Nathanson cohort, Liu cohort, and Riaz cohort. The value of *I* ^2^ represented the heterogeneity level as follows: low (*I* ^2^ < 25%), moderate (*I* ^2^ = 25%–75%), or high (*I* ^2^ > 75%). A random‐effects model was applied for this meta‐analysis. The hazard ratios in each cohort are presented and the horizontal lines indicate the 95% confidence intervals. Melanoma patients in the Nathanson cohort received anti‐CTLA4 therapy, whereas melanoma patients in the Liu cohort and Riaz cohort received anti‐PD‐1 therapy. Patients with gastric cancer in the Kim cohort received anti‐PD‐1 therapy. CR, complete response; DCB, durable clinical benefit; NA, not available; NDB, no durable clinical benefit; PD, progressive disease; PR, partial response; SD, stable disease

We explored the joint prediction power for clinical outcomes of patients treated with ICIs by combining Hh activity with PD‐L1 expression or TMB. Firstly, we divided the population of clinical cohorts into two subgroups with low and high PD‐L1 expression or TMB. Then we explored the association of Hh activity with clinical outcomes of patients treated with ICIs in these subgroups. Strikingly, we found that, for the subgroup with high PD‐L1 expression, the response group harbored consistently lower Hh activity than the no response group in four cohorts (Nathanson cohort: *p* = 0.024; Liu cohort: *p* = 0.002; Riaz cohort: *p* = 0.086; Kim cohort: *p* = 0.030; Figure 5A). However, there was no significance in the subgroup with low PD‐L1 expression (Nathanson cohort: NA; Liu cohort: *p* = 0.263.; Riaz cohort: *p* = 0.725; Kim cohort: *p* = 0.217; Figure 5A). ROC analysis identified that Hh activity was a reliable biomarker for predicting resistance to ICIs in the high PD‐L1 expression subgroup (Nathanson cohort: AUC = 0.929; Liu cohort: AUC = 0.726; Riaz cohort: AUC = 0.721; Kim cohort: AUC = 0.768; Figure 5B) compared to the low PD‐L1 subgroup (Nathanson cohort: AUC = 0.667; Liu cohort: AUC = 0.474; Riaz cohort: AUC = 0.591; Kim cohort: AUC = 0.800; Figure 5B). Furthermore, Hh activity was identified as a risk predictor for OS in the high PD‐L1 subgroup (Nathanson cohort: HR = NA, 95% CI = NA, *p* = 0.032; Liu cohort: HR = 2.98, 95% CI = 1.38–6.43, *p* = 0.003; Riaz cohort: HR = 2.71, 95% CI = 0.85–8.61, *p* = 0.079; Figure 5C,D) in contrast to the low PD‐L1 subgroup (Nathanson cohort: HR = 0.82, 95% CI = 0.22–3.09, *p* = 0.767; Liu cohort: HR = 0.86, 95% CI = 0.43–1.71, *p* = 0.660; Riaz cohort: HR = 0.63, 95% CI = 0.24–1.65, *p* = 0.344; Figure 5C,D). As expected, meta‐analysis validated that patients with high Hh activity had a worse survival in the subgroup with high PD‐L1 expression (HR = 2.89; 95% CI = 1.53–5.49; *p* = 0.001; Figure 5D) in the contrast to the low PD‐L1 expression subgroup (HR = 0.78; 95% CI = 0.46–1.31; *p* = 0.343; Figure 5D). Besides, Hh activity stratified the patients with significantly different PFS in the subgroup with high (HR = 3.19, *p *< 0.001; Figure S10) and low PD‐L1 expression (HR = 1.00, *p* = 0.994; Figure S10). In addition, we investigated the joint prediction by combining Hh activity and TMB. We found that the group with low Hh activity and high TMB showed the most proportion of DCBs, although it was not significant (Nathanson cohort: *p* = 0.12; Liu cohort: *p* = 0.07; Figure S11A). Survival analysis revealed that Hh activity was a risk factor for OS in the subgroup with high TMB in the Liu cohort (HR = 3.35, *p* = 0.012; Figure S11B), while it was not significant in the Nathanson cohort (HR = 1.23, *p* = 0.799; Figure S11B). However, we did not observe the significant association of Hh activity with OS in the subgroup with low TMB (Nathanson cohort: HR = 2.98, *p* = 0.122; Liu cohort: HR = 1.21, *p* = 0.543; Figure S11B). Besides, patients with high Hh activity had a significantly worse PFS both in the subgroup with low (HR = 1.83, *p* = 0.040; Figure S12A) and high TMB (HR = 1.99, *p* = 0.041; Figure S12B). These findings support the combination of Hh activity with PD‐L1 expression to stratify patients receiving ICIs into subgroups with distinct clinical benefits, while combining Hh activity with TMB showed undeniable heterogeneity in different cohorts.

## 4 DISCUSSION

The association of Hh signaling with various aspects of tumor immunobiology has not been systematically studied across multiple cancer types. In addition, the role of Hh signaling in clinical response to ICs therapy remains poorly studied. Herein, based on integrated bioinformatics analysis, we are first to characterize the multifaceted immunosuppressive role of Hh signaling across diverse cancers. Moreover, we identified Hh activity as a negative biomarker for predicting response to ICI treatment and validated the predictive value of Hh activity in multiple independent cohorts. Notably, the combination of Hh activity with PD‐L1 expression showed better predictive efficacy than Hh activity or PD‐L1 expression alone. Patients with low Hh activity and high PD‐L1 expression harbored a higher response rate for ICI therapy and achieved more favorable survival outcomes.

Hh activity has been linked with immunosuppressive TME in several cancers. For instance, Fan et al. found that active Hh signaling could recruit immunosuppressive cells by promoting TGF‐β secretion in BCC.^54^ Hanna et al. demonstrated that Hh signaling inhibitor could reduce immunosuppressive cells, such as M2 macrophages and Treg cells, and increase the number of cytotoxic CD8^+^ T cells and M1 macrophages in breast cancer‐engrafted mice.^18^ In this study, we also observed that tumors with high Hh activity were more inclined to develop an immunosuppressive TME, including decreased CD8^+^ T cells, increased Treg cells, or active TGF‐β signaling, especially in KIRC and LUAD (Figure 3C). Thorsson et al. identified the TGF‐β immune phenotype in a group with mixed tumors from 33 TCGA cancer types that displayed high TGF‐β signaling and high infiltration of CD4^+^ and CD8^+^ T cells.^55^ Similar to the TGF‐β immune phenotype, active TGF‐β signaling was also observed in the group with high Hh activity. Nevertheless, we further found that high Hh activity correlated with decreased CD8^+^ T cells in TME, which was absent in the TGF‐β immune phenotype. Besides, apart from TGF‐β signaling, other features such as active VEGF signaling and ECM organization were recognized as the key promoters for tumor immunosuppression^56^ and were also enriched in the tumors with high Hh activity. Therefore, we considered that the immune phenotypes based on TGF‐β and Hh signaling shared some common features such as activated TGF‐β signaling, but both harbored other distinctive features associated with tumor immunosuppression in TME. Furthermore, several studies suggested a direct regulation of Hh signaling with PD‐L1 expression in BCC^57^ and GC.^19^ These previous findings may partly explain our observation of why the predictive value of Hh activity correlated with PD‐L1 expression.

The identification of predictive biomarkers for ICI therapy has become the central focus of intense research in the era of tumor immunotherapy. PD‐L1 expression and TMB were recognized as effective biomarkers for predicting response to immunotherapy. Besides, in previous transcriptomic studies, a variety of predictive models were also identified as potential immunotherapeutic biomarkers, such as CYT,^38^ GEP,^47^ IFN‐γ,^48^ and APM.^49^ Herein, we conducted correlation analyses to uncover the association between Hh activity and these immunotherapy biomarkers. As shown in Figure S13A, a strong correlation between TME‐related biomarkers was observed in all cancer types, indicating a robust accordance to reflect the immune activity in TME. As expected, Hh activity was negatively correlated with at least one of the TME‐related biomarkers (CYT, GEP, IFN‐γ, and APM) in 71.4%(10/14) TCGA cancer types (CESC, GBM, HNSC, KIRC, LIHC, LUAD, LUSC, OV, SKCM, and STAD), consistent with our previous findings that high Hh activity was associated with an immunosuppressive TME in diverse cancers (Figure 3). On the contrary, Hh activity was positively correlated with PD‐L1 expression at the transcriptional level in 78.6%(11/14) TCGA cancer types (BRCA, CESC, GBM, HNSC, KIRC, KIRP, LIHC, LUAD, LUSC, PAAD, and UCEC). Interestingly, Petty et al. observed that Hh‐induced PD‐L1 on tumor‐associated macrophages suppressed the tumor‐infiltrating CD8^+^ T cell function in TME.^58^ Koh et al. found that Hh signaling‐mediated PD‐L1 promoted infiltration of immunosuppressive MDSCs, leading to the failure response to nivolumab in patient‐derived organoids.^59^ In the subgroup with high PD‐L1 expression, we found that patients with high Hh activity showed dramatically lower response rates to ICIs and had significantly worse clinical outcomes (Figure 5). Taken together, we speculated that high Hh activity might contribute to immunotherapy resistance for those patients with high PD‐L1 expression. As for TMB, we found that the association with Hh activity varied widely across diverse cancers. It was not significant in 57.1%(8/14) of cancers (Figure S13B). However, Hh activity positively correlated with TMB in CESC and was negatively associated with BRCA, LIHC, LUAD, LUSC, and STAD. This demonstrates the different roles of Hh signaling in genomic stability and mutation burden across diverse cancers and warrants more in‐depth exploration in vitro/in vivo.

In clinical practice, using a single biomarker such as PD‐L1 expression could be limited and might incorrectly predict effective response to ICI therapy.^60^, ^61^ Similarly, not all high TMB tumors harbor active immune activity because of the considerable variation across diverse cancers.^38^ This study also evaluated the predictive value of PD‐L1 and TMB in three immunotherapy cohorts. We observed that the single‐biomarker strategy by using PD‐L1 or TMB alone showed unstable prediction efficiency in different populations and undeniable heterogeneity between the independent cohorts (Figures S5 and S6). Interestingly, a combined strategy by integrating Hh activity and PD‐L1 expression, which achieved better predictive power than Hh activity or PD‐L1 expression alone (Figure 5). It could be explained that a composite biomarker might capture the immune status of the TME more effectively than a single biomarker.^62^ In addition to those routine biomarkers used in clinical practice, numerous models have also been established to predict response to ICI therapy in previous studies. For instance, Jiang et al. developed a model named Tumor Immune Dysfunction and Exclusion (TIDE) to predict cancer immunotherapy response.^63^ In this study, we further compared the difference in predictive power between Hh activity and TIDE. We found that Hh activity showed more effective predictive power for resistance to ICI response than TIDE in four independent cohorts (AUC: 0.817 vs. 0.558 in the Nathanson cohort; 0.590 vs. 0.554 in the Liu cohort; 0.733 vs. 0.642 in the Riaz cohort; 0.725 vs. 0.604 in the Kim cohort; Figure S14; Figure 4B). In summary, compared with those biomarkers, Hh activity harbored several advantages as follows: (1) Under the background that identification of positive predictive biomarkers has become a hot spot of intense research, Hh activity serves as a negative predictive biomarker, which can exclude a considerable proportion of non‐responders. Therefore, Hh activity might be considered as a supplementary index for its negative predictive value in tumor immunotherapy. (2) This study developed a combined strategy by integrating Hh activity and PD‐L1 expression, which offered a more comprehensive immune status of the TME and provided more predictive efficiency than a single biomarker alone. (3) Hh activity reflects the activation status of the Hh signaling pathway, which has been recognized as a hallmark signaling in various cancers.^64^ Encouragingly, a series of Hh signaling inhibitors have been developed in preclinical studies or clinical trials,^65^ which provide substantial hope for targeting Hh signaling in diverse cancers to overcome tumor immunotherapy resistance in the future.

In recent years, resistance to mono ICI therapy remains a great challenge for a non‐negligible number of patients with metastatic cancers.^66^ Schadendorf et al. reported that nearly 20% of melanoma patients treated with ipilimumab show DCBs and long‐term survival.^67^ Ribas et al. reported that the 3‐year response rate was only 33% for melanoma patients treated with pembrolizumab.^68^ Those patients who had limited or no response to mono ICI therapy may become suitable candidates for the combination of ICI therapy with other therapeutic interventions, such as chemoradiotherapy^69^ and target therapy.^70^ These therapies may convert tumors from immune deserted/excluded type to immune inflamed type, leading to the enhanced anti‐tumor response after the administration of ICI therapy.^71^ However, the shallow understanding of potential targets involved in the immunotype transformation from “cold” to “hot” has limited the development of precise combination treatment. The predictive biomarker identified in this study was based on the Hh signaling, recognized as a canonical oncogenic signaling and a therapeutic target.^72^, ^73^ Otsuka et al. reported that tumor regression induced by Hh signaling inhibitor was accompanied by recruitment of cytotoxic T cells into the TME in BCC,^21^ indicating the great potential of Hh signaling as a critical target in combination treatments. Encouragingly, three clinical trials comparing the treatment efficiency between the mono ICI group or the combined group (ICI plus Hh inhibitors) in metastatic BCC patients will provide evidence of whether combination treatments serve as a better strategy than mono ICI strategy in BCC (NCT03521830; NCT03132636; NCT02690948). Our study found that metastatic melanoma and GC patients with high Hh activity developed a limited response rate to ICI therapy, whereas patients with low Hh activity exhibited better therapeutic outcomes. Therefore, we speculate that the combination treatments targeting Hh signaling and immune checkpoints may synergistically increase the efficacy and durability of the therapeutic outcomes across diverse cancers.

It is worth noting that this study has several limitations. First, our study investigated the relationship between Hh signaling and TME using bioinformatic analysis; therefore, further in vitro/in vivo experiments need to be conducted for substantiation. Second, the inconsistent prognostic value of Hh activity alone was observed between the individual cohorts and meta‐cohorts. The small sample size and a limited number of studies might contribute to the negative results in the sensitivity analysis. The patient samples in the cohorts were treated differently and at different time points, which might cause unavoidable bias in this study. Therefore, the prognostic value of Hh activity in patients receiving ICI therapy requires more validation in large‐scale clinical cohorts and prospective clinical trials. Third, a total of 14 cancer types were enrolled in this pan‐cancer analysis, but the predictive value of Hh activity for response to ICI therapy was explored in the clinical cohorts with metastatic melanoma and GC. Therefore, the role of Hh activity in tumor immunosuppression and ICI therapy could be explored and validated in more cancer types. In addition, we did not distinguish the tumor samples from primary and metastatic lesions in the TCGA‐SKCM, which might bring potential bias into this study. Besides, considering the expensive cost and intense time from RNA‐seq, the method of estimating Hh activity needs to be simplified in clinical practice.

In conclusion, our study highlights that increased Hh activity correlated with multiple immunosuppressive characteristics in the TME of diverse cancers. Moreover, Hh activity was a predictive biomarker for resistance to ICI therapy in patients with metastatic cancer. Furthermore, combination with PD‐L1 expression was able to better predict clinical outcomes than a single biomarker. These findings deserve experimental validation and prospective investigation in the future to assist oncologists in precise treatment recommendations for cancer patients.

## CONFLICTS OF INTEREST

The authors declare that they have no conflict of interests.

## AUTHORS' CONTRIBUTIONS

Lisong Teng and Haiyong Wang conceived and designed the study. Junjie Jiang and Yongfeng Ding collected data, performed data analysis, and wrote the manuscript. Junjie Jiang and Yanyan Chen drew figures. Yongfeng Ding and Jun Lu performed literature search and RT‐PCR experiments. Yiran Chen, Guanghao Wu, and Nong Xu were involved in data interpretation and critically reviewing the manuscript. All authors read and approved the final manuscript. All authors read and approved the final manuscript.

## ETHICS STATEMENT

This study involving human samples were reviewed and approved by the Research Ethics Board of the First Affiliated Hospital of Zhejiang University.

## Supporting information

Fig S1‐14

Table S1‐4

## Data Availability

TCGA datasets used in this study were downloaded from the cBioPortal database (https://www.cbioportal.org/). Four immunotherapy datasets were downloaded from the Supporting Information of the original articles (Nathanson cohort: 10.1158/2326‐6066.CIR‐16‐0019; Liu cohort: 10.1038/s41591‐020‐0975‐4; Riaz cohort: 10.1016/j.cell.2017.09.028; Kim cohort: 10.1038/s41591‐018‐0101‐z). The Hedgehog signaling geneset was downloaded from the Molecular Signatures Database (https://www.gsea‐msigdb.org/gsea/msigdb). The profile of tumor‐infiltrating immune cells fractions in TCGA cancers was downloaded from The Cancer Immunome Atlas database (https://tcia.at/).
