# Supplementary material for: Pan‐cancer analyses reveal that increased Hedgehog activity correlates with tumor immunosuppression and resistance to immune checkpoint inhibitors
Source: Cancer Med. 2021 Nov 28;11(3):847–63. doi: 10.1002/cam4.4456 (PMC8817099; doi:10.1002/cam4.4456)
Supplement: Supplementary file 1 — Fig S1‐14 [file CAM4-11-847-s001.pdf]

**Supplementary Figure**

**Figure S1. Forest plot of multivariate Cox regression analysis showing the association between Hedgehog activity and overall survival across 14 cancer types adjusted by age.** The median value of Hh activity was adopted as the threshold for the groups with low and high Hh activity. The hazard ratios are presented and the horizontal lines indicate the 95% confidence intervals. Hh, Hedgehog; HR, hazard ratio; CI, confidence interval.

**Figure S2. Association of Hh activity with immune cells in diverse cancer.** The 22 types of immune cells were estimated by CIBERSORT. The comparison of the average abundance of immune cells was conducted between the group with low and high Hh activity in each cancer type. Each point represents each cancer type. The heatmap indicates the P value of each comparison. Rows show 14 cancer types and columns show 22 types of immune cells. Annotation bar indicates the P value of each comparison.

**Figure S3. Boxplots showing the comparison of TGF-β signaling and Wnt signaling activity between the groups with low and high Hh activity across 14 cancer types.** The activity of TGF-β signaling and Wnt signaling is estimated by ssGSEA analysis.

**Figure S4. Summary graphic showing the role of Hedgehog activity in tumor immune evasion.**

**Figure S5. Association between PD-L1 expression and clinical outcomes in the patients treated with ICIs. (A)** Comparison of the clinical benefit rate between the groups with low and high PD-L1 expression in the Nathanson cohort, Liu cohort, Riaz cohort, and Kim cohort. **(B)** Kaplan-Meier curves showing the association of PD-L1 expression with OS in the Nathanson cohort (left panel), Liu cohort (middle panel), and Riaz cohort (right panel). **(C)** Forest plot showing meta analysis for the prognostic value of PD-L1 expression in the Nathanson cohort, Liu cohort, and Riaz cohort. HR, hazard ratio; CI, confidence interval; OS, overall survival.

**Figure S6. Association between TMB and clinical outcomes in the patients treated with ICIs. (A)** Comparison of clinical benefit rate between the groups with low and high TMB in the Nathanson cohort (left panel) and Liu cohort (right panel). **(B)** Kaplan–Meier curves showing the association of TMB with OS in the Nathanson cohort (left panel) and Liu cohort (right panel). TMB, tumor mutation burden.

**Figure S7. Sensitivity analysis and publication bias analysis. (A)** Sensitivity analysis for overall survival. **(B-C)** Assessment of publication bias for overall survival using Begg's test **(B)** and Egger's test **(C)**.

**Figure S8. Association between transcriptional expression of two Hh-related genes (*GLI1* and *SHH*) and clinical outcomes of patients treated with ICIs.** (A) Histograms showing the comparison of *GLI1* and *SHH* between the groups with PD/SD and PR/CR. (B) Kaplan-Meier curves showing the association of *GLI1* and *SHH* with OS. A total of 10 tumor samples of GC patients were obtained from the First Affiliated Hospital of Zhejiang University. 8 samples were collected from GC patients receiving neoadjuvant immunotherapy, while 2 samples were collected from GC patients receiving adjuvant immunotherapy. Gene transcription expression levels of *GLI1* and *SHH* were detected using qRT-PCR. PD, progressive disease; SD, stable disease; PR, partial response; CR, complete response; OS, overall survival; GC, gastric cancer.

**Figure S9. Kaplan-Meier curves of progression-free survival in the Liu cohort grouped by Hh**

**activity (A), PD-L1 expression (B), and TMB (C), respectively.** PFS, progression-free survival.

**Figure S10. Association between Hedgehog activity and progression-free survival in the subgroups stratified by PD-L1 expression.** Kaplan-Meier curves show the prognostic value of Hh activity in the subgroup with low **(A)** and high **(B)** PD-L1 expression.

**Figure S11. Association between Hedgehog activity and clinical outcomes in the subgroups stratified by TMB. (A)** Association between Hh activity and clinical benefit from ICIs therapy in the subgroups stratified by TMB in the Nathanson cohort (upper panel) and Liu cohort (lower panel). **(B)** Kaplan-Meier curves showing the association between Hh activity and OS in the subgroups stratified by TMB in the Nathanson cohort (left panel) and Liu cohort (right panel). The median value of TMB is adopted as the cutoff value. $HH^{high}$, high Hh activity group; $HH^{low}$, low Hh activity group.

**Figure S12. Association between Hedgehog activity and progression-free survival in the subgroups stratified by TMB.** Kaplan-Meier curves show the prognostic value of Hh activity in the subgroup with low **(A)** and high **(B)** TMB.

**Figure S13. Correlation analysis between Hh activity with predictive biomarkers for immunotherapy. (A)** PD-L1 expression, CYT, GEP, IFN-γ, and APM; **(B)** TMB. CYT, cytolytic activity; GEP, T cell–inflamed gene expression profile; IFN-γ, IFN-γ signature; APM, MHC class I antigen presenting machinery expression; TMB, tumor mutation burden.

**Figure S14. ROC curves for predicting resistance to ICIs therapy by TIDE in the Nathanson cohort (orange), Liu cohort (green), Riaz cohort (blue), and Kim cohort (purple).** TIDE, Tumor Immune Dysfunction and Exclusion; ROC, receiver operating characteristic.

**Supplementary Figure**

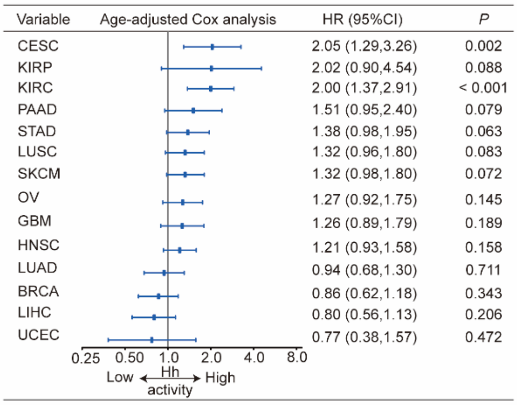


**Figure S1. Forest plot of multivariate Cox regression analysis showing the association between Hedgehog activity and overall survival across 14 cancer types adjusted by age.** The median value of Hh activity was adopted as the threshold for the groups with low and high Hh activity. The hazard ratios are presented and the horizontal lines indicate the 95% confidence intervals. Hh, Hedgehog; HR, hazard ratio; CI, confidence interval.

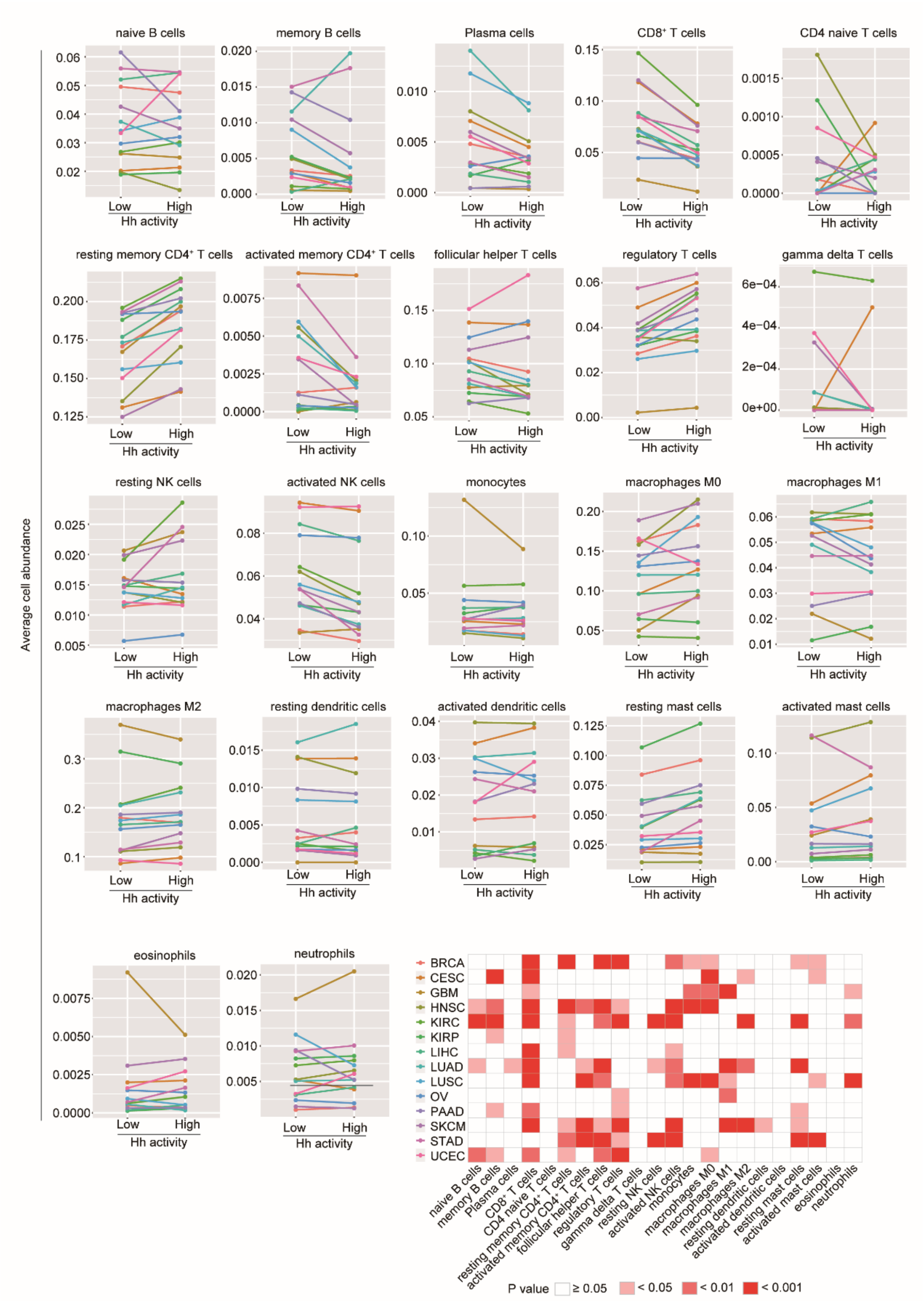


**Figure S2. Association of Hh activity with immune cells in TME across diverse cancer types.** The 22 types of immune cells were estimated by CIBERSORT. The average abundance of immune cells was compared between the group with low and high Hh activity in each cancer type. Each point represents the average cell abundance of each cancer type. The heatmap indicates the P value of each comparison. Rows show 14 cancer types and columns show 22 types of immune cells. Annotation bar indicates the P value of each comparison.

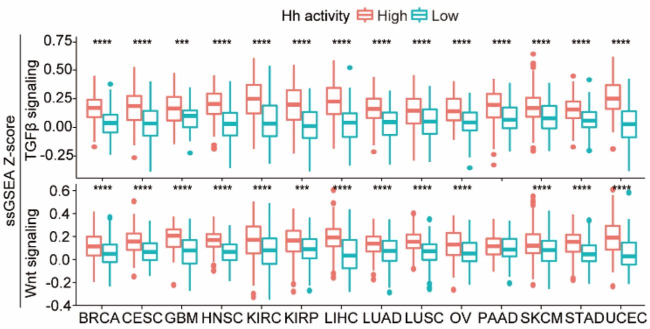


**Figure S3. Boxplots showing the comparison of TGF-β signaling and Wnt signaling activity between the groups with low and high Hh activity across 14 cancer types.** The activity of TGF-β signaling and Wnt signaling is estimated by ssGSEA analysis.

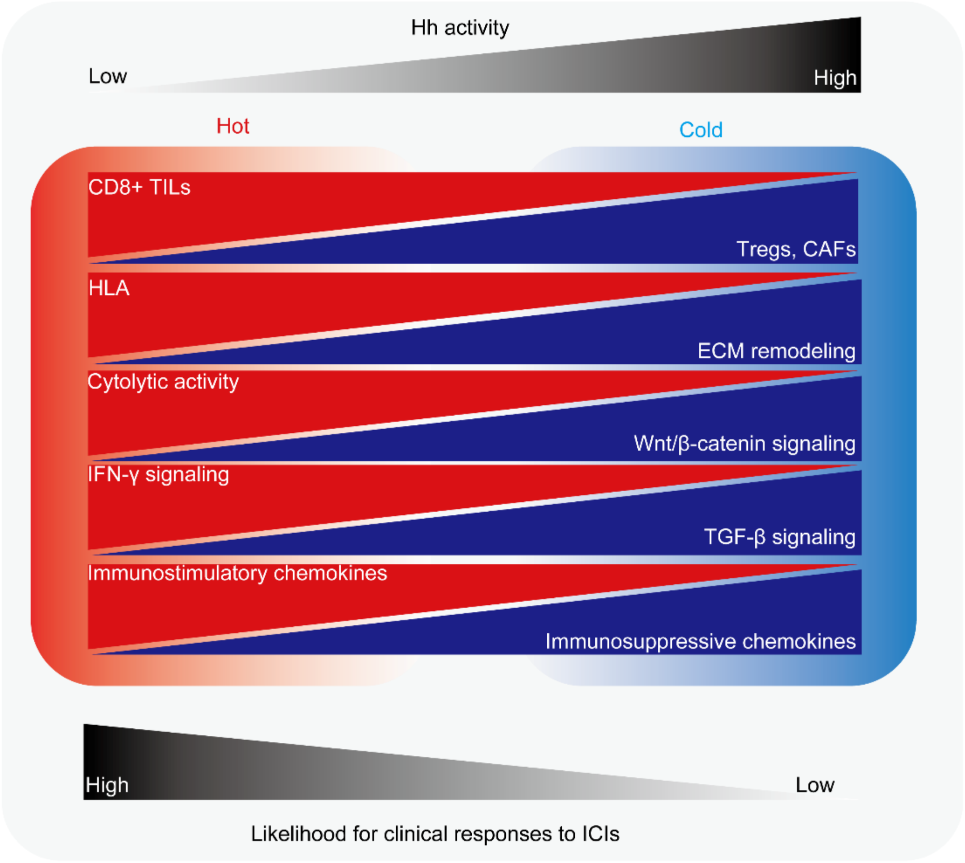


**Figure S4. Summary graphic showing the role of Hedgehog activity in tumor immune evasion.**

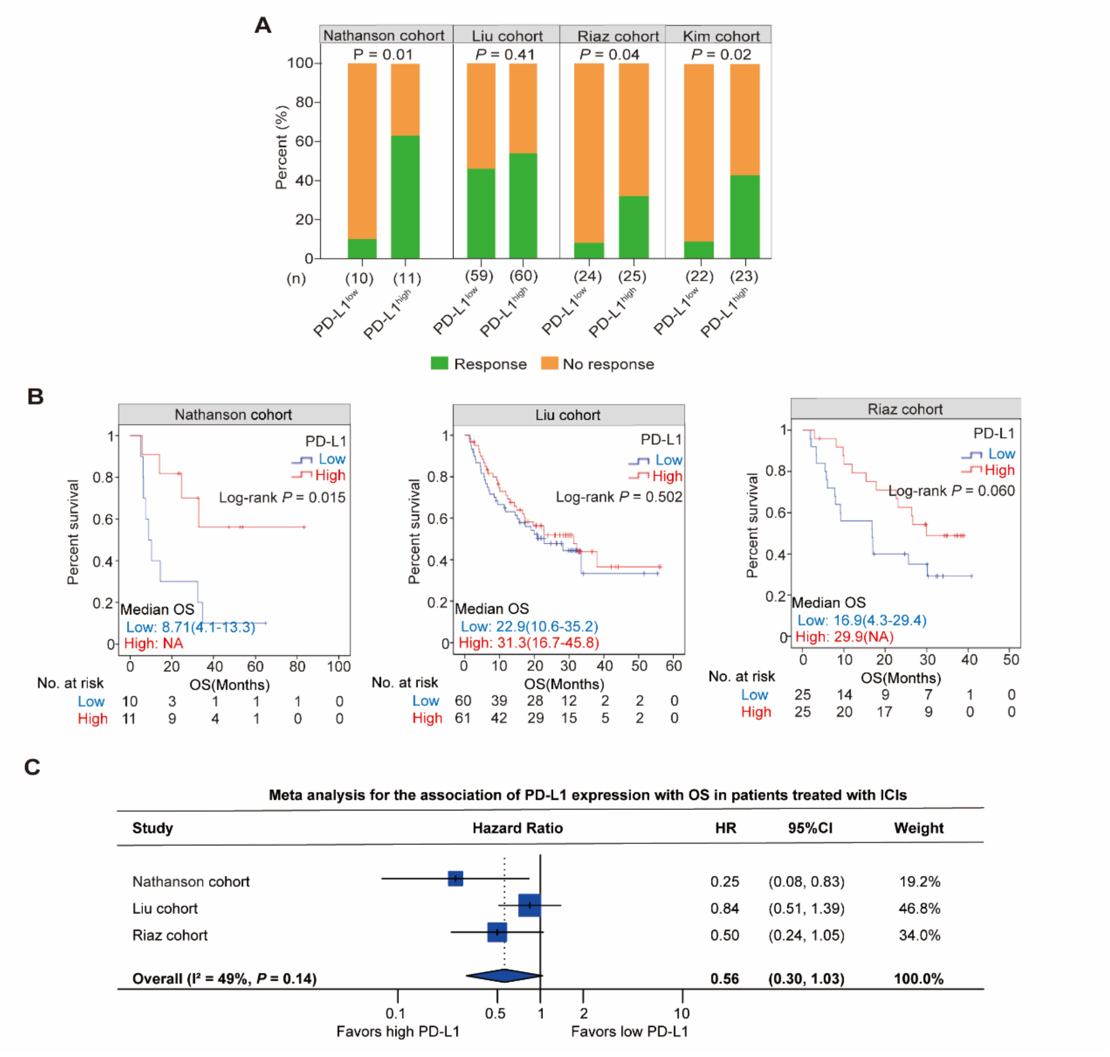


**Figure S5. Association between PD-L1 expression and clinical outcomes in the patients treated with ICIs. (A)** Comparison of the clinical benefit rate between the groups with low and high PD-L1 expression in the Nathanson cohort, Liu cohort, Riaz cohort, and Kim cohort. **(B)** Kaplan-Meier curves showing the association of PD-L1 expression with OS in the Nathanson cohort (left panel), Liu cohort (middle panel), and Riaz cohort (right panel). **(C)** Forest plot showing meta analysis for the prognostic value of PD-L1 expression in the Nathanson cohort, Liu cohort, and Riaz cohort. HR, hazard ratio; CI, confidence interval; OS, overall survival.

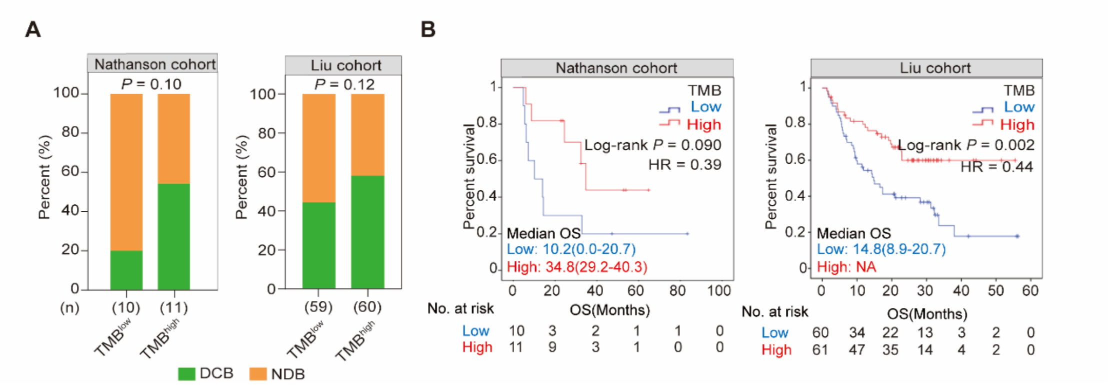


**Figure S6. Association between TMB and clinical outcomes in the patients treated with ICIs. (A)** Comparison of clinical benefit rate between the groups with low and high TMB in the Nathanson cohort (left panel) and Liu cohort (right panel). **(B)** Kaplan–Meier curves showing the association of TMB with OS in the Nathanson cohort (left panel) and Liu cohort (right panel). TMB, tumor mutation burden.

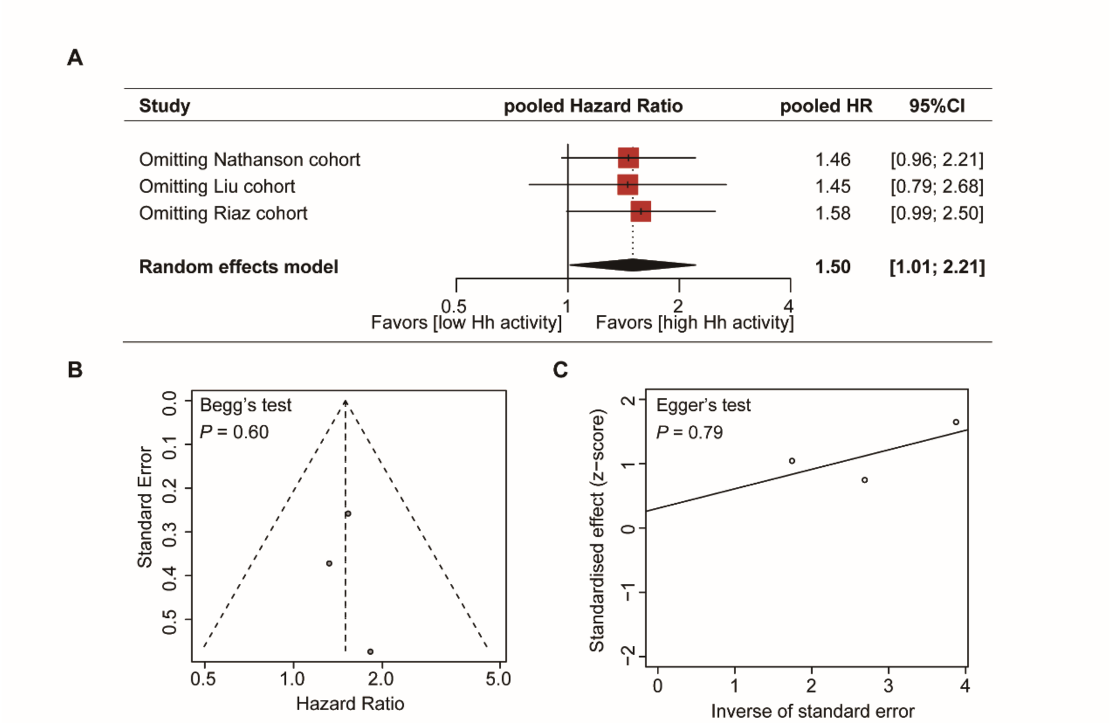


**Figure S7. Sensitivity analysis and publication bias analysis. (A)** Sensitivity analysis for overall survival. **(B-C)** Assessment of publication bias for overall survival using Begg's test **(B)** and Egger's test **(C)**.

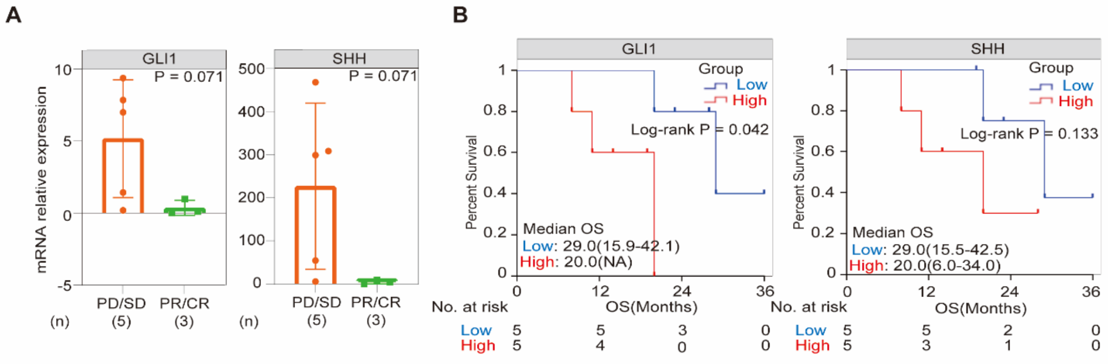


**Figure S8. Association between transcriptional expression of two Hh-related genes (*GLI1* and *SHH*) and clinical outcomes of patients treated with ICIs.** (A) Histograms showing the comparison of *GLI1* and *SHH* between the groups with PD/SD and PR/CR. (B) Kaplan-Meier curves showing the association of *GLI1* and *SHH* with OS. A total of 10 patient samples were collected from our institution for validation experiments. 8 samples were collected from patients receiving neoadjuvant immunotherapy, while 2 samples were collected from patients receiving adjuvant immunotherapy. Gene transcription expression level of *GLI1* and *SHH* were detected using qRT-PCR. PD, progressive disease; SD, stable disease; PR, partial response; CR, complete response. OS, overall survival.

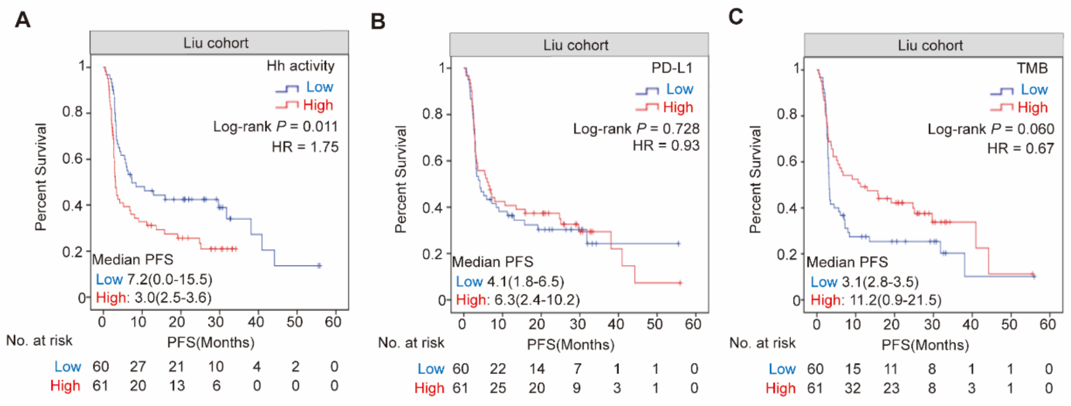


**Figure S9. Kaplan-Meier curves of progression-free survival in the Liu cohort grouped by Hh activity (A), PD-L1 expression (B), and TMB (C), respectively.** PFS, progression-free survival.

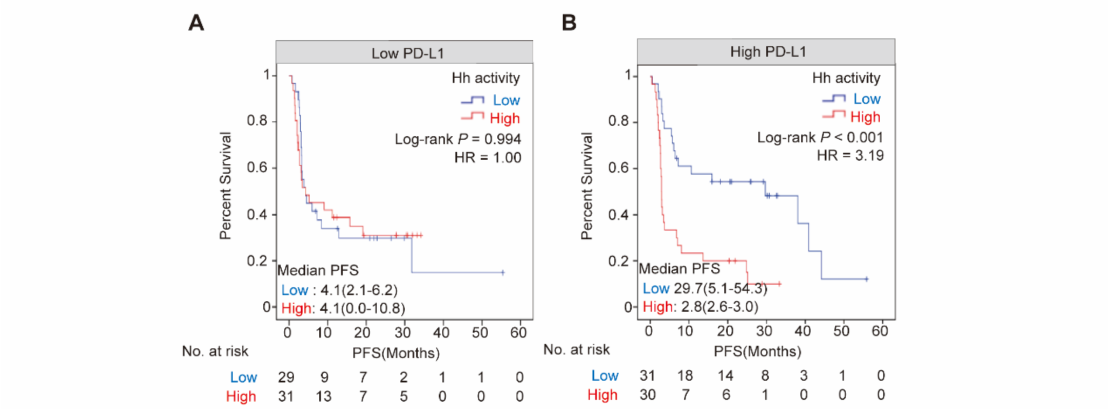


**Figure S10. Association between Hedgehog activity and progression-free survival in the subgroups stratified by PD-L1 expression.** Kaplan-Meier curves show the prognostic value of Hh activity in the subgroup with low **(A)** and high **(B)** PD-L1 expression.

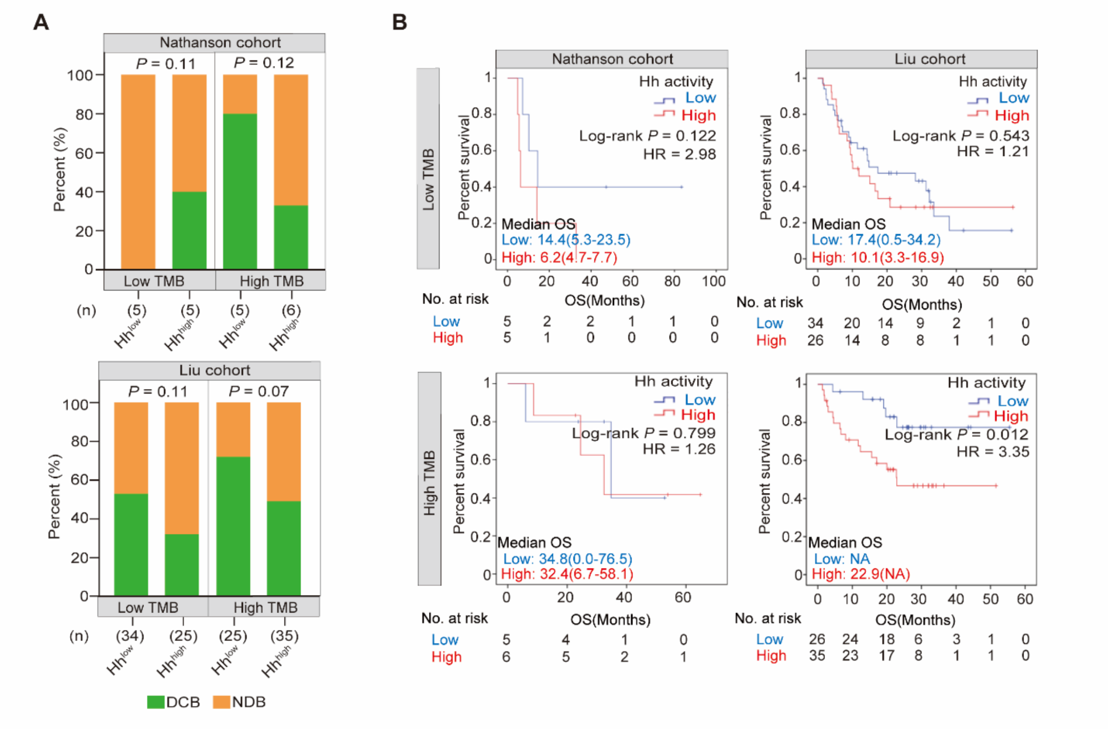


**Figure S11. Association between Hedgehog activity and clinical outcomes in the subgroups stratified by TMB. (A)** Association between Hh activity and clinical benefit from ICIs therapy in the subgroups stratified by TMB in the Nathanson cohort (upper panel) and Liu cohort (lower panel). **(B)** Kaplan-Meier curves showing the association between Hh activity and OS in the subgroups stratified by TMB in the Nathanson cohort (left panel) and Liu cohort (right panel). The median values of TMB is adopted as the cutoff value. $HH^{high}$, high Hh activity group; $HH^{low}$, low Hh activity group.

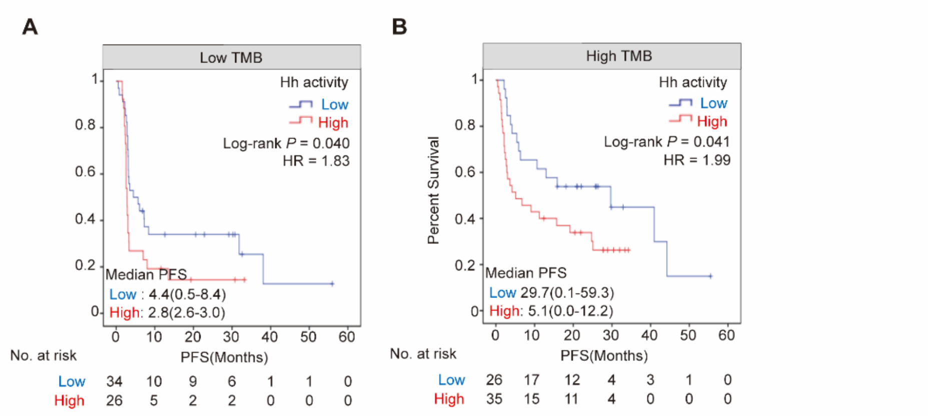


**Figure S12. Association between Hedgehog activity and progression-free survival in the subgroups stratified by TMB.** Kaplan-Meier curves show the prognostic value of Hh activity in the subgroup with low **(A)** and high **(B)** TMB.

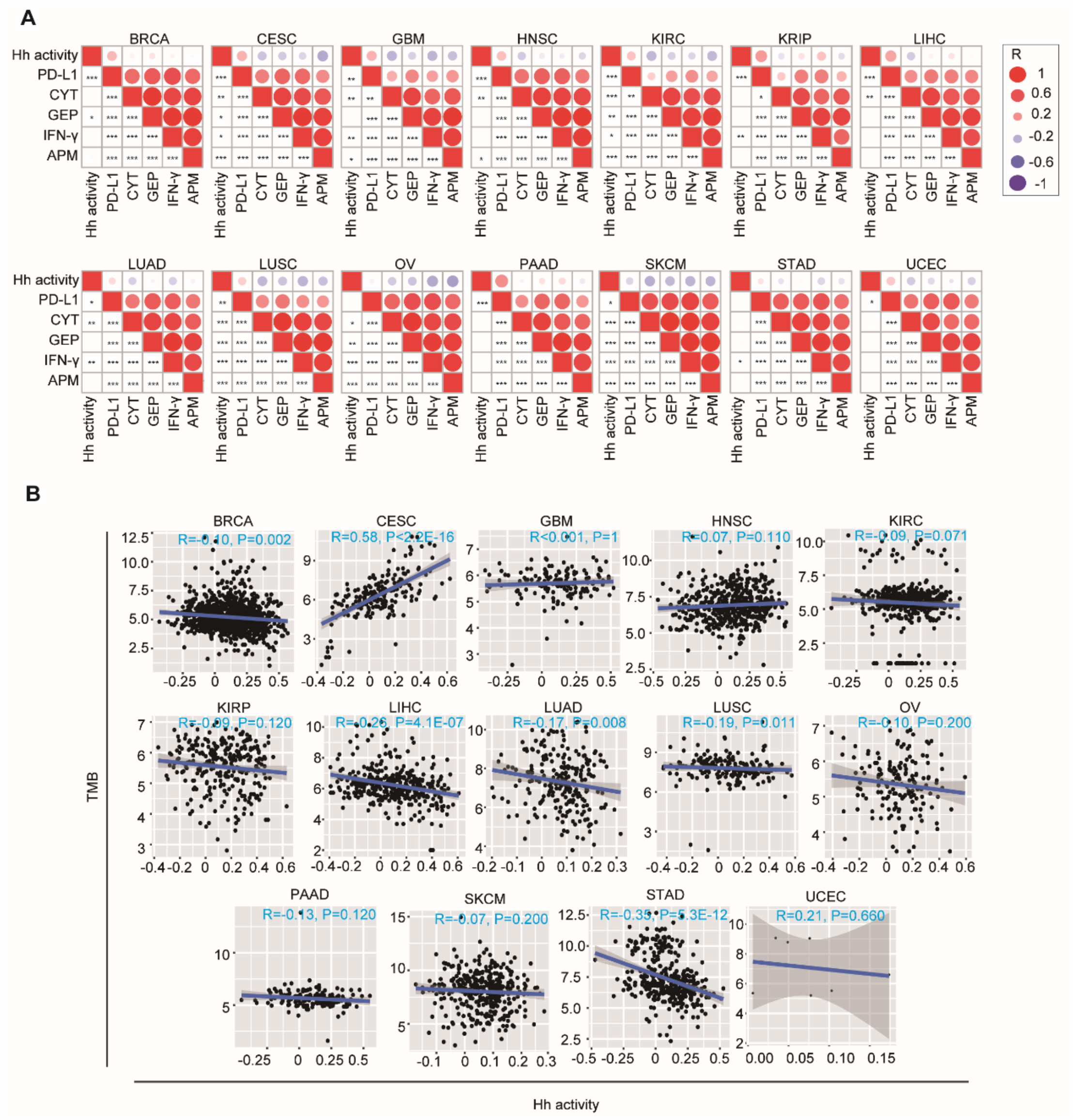


**Figure S13. Correlation analysis between Hh activity with predictive biomarkers for immunotherapy. (A)** PD-L1 expression, CYT, GEP, IFN-γ, and APM; The Spearman's correlation coefficient was shown as the circle at the upper triangle and the statistical significance was shown at the lower triangle. Annotation bar indicates the value of correlation coefficient. Red and blue represents the positive and negative correlation, respectively. $^{*}P < 0.05$, $^{**}P < 0.01$, $^{***}P < 0.001$. **(B)** TMB. Spearman's correlation analyses were performed between Hh activity and TMB. CYT, cytolytic activity; GEP, T cell–inflamed gene expression profile; IFN-γ, IFN-γ signature; APM, MHC class I antigen presenting machinery expression; TMB, tumor mutation burden.

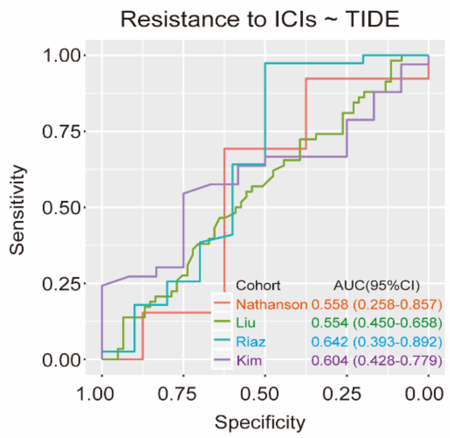


**Figure S14. ROC curves for predicting resistance to ICIs therapy by TIDE in the Nathanson cohort (orange), Liu cohort (green), Riaz cohort (blue), and Kim cohort (purple).** TIDE, Tumor Immune Dysfunction and Exclusion; ROC, receiver operating characteristic.
