# Supplementary material for: Pan‐cancer analyses reveal that increased Hedgehog activity correlates with tumor immunosuppression and resistance to immune checkpoint inhibitors
Source: Cancer Med. 2021 Nov 28;11(3):847–63. doi: 10.1002/cam4.4456 (PMC8817099; doi:10.1002/cam4.4456)
Supplement: Supplementary file 2 — Table S1‐4 [file CAM4-11-847-s002.pdf]

Table S1. Baseline characteristics of patients treated with ICIs

Characteristic	Nathanson cohort (N = 21)	Liu cohort (N = 121)	Riaz cohort (N = 50)	Kim cohort (N = 61)
**Cancer type**	melanoma	melanoma	melanoma	gastric cancer
**Age – yrs**				
Median	58	NA	NA	57
Range	(33, 90)	NA	NA	(26, 78)
**Gender - no. (%)**				
Female	11(52.4)	50(41.3)	NA	18(29.5)
Male	10(47.6)	71(58.7)	NA	43(70.5)
**Drug received - no. (%)**				
Ipilimumab	20(95.2)	0(0)	0(0)	0(0)
Tremelimumab	1(4.8)	0(0)	0(0)	0(0)
Nivolumab	0(0)	51(42.1)	50(100)	0(0)
Pembrolizumab	0(0)	70(57.9)	0(0)	61(100)
**Sample collection - no. (%)**				
Before treatment	7(33.3)	120(99.2)	42(85.7)	61(100)
After treatment	14(66.7)	1(0.8)	7(14.3)	0(0)
**Previous ICIs therapy**				
Yes	0(0)	NA	29(59.2)	0(0)
No	21(100)	NA	20(40.8)	61(100)
**Best objective response - no. (%)**				
CR	NA	16(13.2)	3(6.0)	3(4.9)
PR	NA	31(25.6)	7(14.0)	12(19.7)
SD	NA	16(13.2)	18(36.0)	20(32.8)
PD	NA	56(46.3)	21(42.0)	22(36.1)
MR	NA	2(1.7)	0(0)	0(0)
NE	NA	0(0)	1(2.0)	4(6.6)
**Overall survival - mos**				
Median	23.7	18.1	22.8	NA
Range	(4.8, 83.4)	(1.3, 56.4)	(1.9, 40.9)	NA
**Progression-free survival - mos**				
Median	NA	5.4	NA	NA
Range	NA	(0.4, 56.0)	NA	NA
**Durable clinical benefit - no. (%)**				
DCB	8(38.1)	61(50.4)	10(20.0)	NA
NDB	13(61.9)	58(47.9)	39(78.0)	NA
NA	0(0)	2(1.7)	1(2.0)	NA
**RNA-seq sample with annotated clinical information- no. (%)**				
Available	21(100)	119(98.3)	49(98.0)	45(73.8)
Not available	0(0)	2(1.7)	1(2.0)	16(26.2)

Abbreviations: ICIs, immune checkpoint inhibitors; CR, complete response; PR, partial response; SD, stable disease; PD, progressive disease; NE, not evaluable; DCB, durable clinical benefit; NDB, no durable clinical benefit; yrs, years; mos, months; NA, not available.

Table S2. The detail of Hedgehog-related genes in the HALLMARK HEDGEHOG SIGNALING geneset

Gene ID	Entrez Gene ID	Description
ACHE	43	Acetylcholinesterase
AMOT	154796	Angiomotin
CDK5R1	8851	Cyclin dependent kinase 5 regulatory subunit
CDK6	1021	Cyclin dependent kinase 6
CELSR1	9620	Cadherin EGF LAG seven-pass G-type receptor 1
CNTFR	1271	Ciliary neurotrophic factor receptor mediator
CRMP1	1400	Collapsin response protein 1
DPYSL2	1808	Dihydropyrimidinase like 2
ETS2	2114	ETS proto-oncogene 2
GLI1	2735	GLI family zinc finger 1
GPR56	9289	Adhesion G protein-coupled receptor G1
HEY1	23462	Hes related family bHLH transcription factor with YRPW motif 1
HEY2	23493	Hes related family bHLH transcription factor with YRPW motif 2
L1CAM	3897	L1 cell adhesion molecule
LDB1	8861	LIM domain binding 1
MYH9	4627	Myosin heavy chain 9
NF1	4763	Neurofibromin 1
NKX6-1	4825	NK6 homeobox 1
NRCAM	4897	Neuronal cell adhesion molecule
NRP1	8829	Neuropilin 1
NRP2	8828	Neuropilin 2
OPHN1	4983	Oligophrenin 1
PLG	5340	Plasminogen
PML	5371	Promyelocytic leukemia
PTCH1	5727	Patched 1
RASA1	5921	RAS p21 protein activator 1
RTN1	6252	Reticulon 1
SCG2	7857	Secretogranin II
SHH	6469	Sonic hedgehog signaling molecule
SLIT1	6585	Slit guidance ligand 1
THY1	7070	Thy-1 cell surface antigen
TLE1	7088	TLE family member 1
TLE3	7090	TLE family member 3
UNC5C	8633	Unc-5 netrin receptor C
VEGFA	7422	Vascular endothelial growth factor A
VLDLR	7436	Very low density lipoprotein receptor

Table S3. The spearman's correlation between Hh activity and Hedgehog-related genes across diverse cancers

Gene	BRCA	CESC	GBM	HNSC	KIRC	KIRP	LIHC	LUAD	LUSC	OV	PAAD	SKCM	STAD	UCEC
SHH	0.012	-0.015	0.178	0.106	0.117	0.324	0.291	0.254	0.282	0.118	-0.006	0.045	0.062	0.208
PTCH1	0.354	0.151	0.293	0.151	0.564	0.559	0.454	0.429	0.349	0.253	0.315	0.329	0.475	0.403
NRCAM	0.327	0.245	0.287	0.217	0.156	0.162	0.192	0.313	0.360	0.351	0.335	0.297	0.267	0.452
NRP1	0.578	0.358	0.321	0.448	0.645	0.586	0.713	0.381	0.218	0.362	0.493	0.188	0.469	0.331
SCG2	0.398	0.164	0.315	0.292	0.176	0.222	0.420	0.295	0.238	0.295	0.316	0.087	0.341	0.201
AMOT	0.167	-0.105	-0.099	0.012	0.363	0.377	0.313	0.125	0.033	0.303	0.276	0.113	0.235	0.241
UNC5C	0.446	0.219	0.317	0.418	0.638	0.451	0.516	0.536	0.292	0.230	0.416	0.179	0.495	0.285
ADGRG1	0.157	0.227	0.312	0.376	0.201	0.269	0.439	0.269	0.343	0.086	0.176	0.082	0.248	0.337
HEY1	0.286	0.180	0.004	0.070	0.262	0.049	0.169	0.158	0.157	0.140	0.101	0.047	0.380	0.145
GLI1	0.266	0.282	0.003	0.348	0.246	0.255	0.336	0.424	0.452	0.397	0.340	0.274	0.468	0.128
THY1	0.215	0.117	0.113	0.193	0.189	0.021	0.202	0.184	0.200	0.221	0.124	0.029	0.350	-0.015
SLIT1	0.090	-0.036	0.331	-0.154	0.026	-0.143	0.149	0.156	-0.026	0.298	0.306	0.149	0.251	-0.025
CDK6	0.404	0.440	0.266	0.502	0.304	0.367	0.290	0.220	0.355	0.430	0.357	0.175	0.155	0.298
HEY2	0.250	0.134	0.048	0.127	0.257	-0.030	-0.033	0.318	0.235	-0.016	0.134	-0.081	0.324	-0.021
NRP2	0.443	0.306	0.344	0.531	0.350	0.273	0.537	0.361	0.397	0.285	0.294	0.206	0.515	0.621
TLE3	-0.054	0.289	0.315	0.096	0.248	0.255	0.207	0.303	0.289	0.251	0.028	0.118	0.435	0.180
TLE1	0.060	0.037	0.008	0.013	-0.009	0.086	-0.051	-0.036	0.173	0.138	-0.024	0.082	-0.058	0.027
L1CAM	0.284	0.052	0.323	0.269	-0.060	0.144	0.223	0.103	0.159	0.275	0.171	0.078	0.320	0.094
PLG	0.059	0.032	0.036	-0.016	0.158	0.007	-0.035	0.065	-0.013	0.131	0.055	-0.075	0.001	0.111
NKX6-1	0.300	0.265	0.274	0.246	-0.074	-0.044	0.096	0.061	0.296	0.195	0.307	0.339	0.198	0.122
NF1	0.340	0.290	0.322	0.502	0.607	0.638	0.536	0.440	0.323	0.396	0.566	0.282	0.341	0.520
RASA1	0.289	0.523	0.325	0.425	0.452	0.558	0.531	0.483	0.304	0.473	0.551	0.305	0.284	0.574
ETS2	0.397	0.164	0.242	0.230	0.324	0.213	0.210	0.230	0.254	0.295	0.200	0.121	0.204	0.387
RTN1	0.095	0.114	-0.050	0.099	0.066	0.239	0.300	0.248	-0.008	0.120	0.297	-0.051	0.312	0.207
CRMP1	0.405	0.330	0.270	0.302	0.368	0.354	0.386	0.420	0.456	0.443	0.404	0.243	0.506	0.286
MYH9	0.420	0.474	0.476	0.644	0.428	0.502	0.528	0.503	0.499	0.328	0.223	0.102	0.438	0.528
VEGFA	0.221	0.416	0.362	0.188	0.281	0.123	0.309	0.152	0.228	0.220	0.335	0.115	-0.015	0.326
CELSR1	0.055	0.342	0.374	0.612	0.357	0.531	0.491	0.405	0.479	0.296	0.166	0.136	0.328	0.404
CNTFR	0.268	0.243	0.086	0.123	0.054	0.024	0.155	0.250	0.215	0.278	0.133	0.304	0.206	0.153
ACHE	0.130	-0.070	0.184	0.043	-0.202	-0.208	0.106	0.055	-0.085	0.166	-0.123	0.025	-0.123	-0.050

PML	0.193	-0.050	0.235	0.118	-0.041	0.037	0.312	0.175	0.164	0.071	-0.020	-0.071	0.184	0.039
CDK5R1	0.283	0.532	0.252	0.466	0.120	0.006	0.377	0.200	0.448	0.533	0.371	0.158	0.242	0.474
VLDLR	0.393	0.388	0.442	0.281	0.308	0.526	0.452	0.264	0.380	0.290	0.473	0.391	0.518	0.520
OPHN1	0.260	0.098	0.082	0.265	0.529	0.479	0.301	0.185	0.130	0.197	0.375	0.024	0.383	0.323
LDB1	0.124	0.433	0.268	0.329	0.222	0.394	0.585	0.251	0.177	0.364	0.249	0.172	0.457	0.499
DPYSL2	0.478	0.110	0.384	0.216	0.508	0.419	0.451	0.458	0.211	0.490	0.258	0.372	0.449	0.337

Table S4. Distinctive pathways enriched in the tumors with different Hh activity across diverse cancers

Hh activity	Pathway	FDR													
		BRCA	CESC	GBM	HNSC	KIRC	KIRP	LIHC	LUAD	LUSC	OV	PAAD	SKCM	STAD	UCEC
Low	REACTOME_APC_C:CDH1_MEDIATED_DEGRADATION_OF_CDC20_AND_OTHER_APC_C:CDH1_TARGETED_PROTEINS_IN_LATE_MITOSIS_EARLY_G1	< 0.001	< 0.001	< 0.001	< 0.001	< 0.001	< 0.001	< 0.001	< 0.001	< 0.001	< 0.001	< 0.001	< 0.001	< 0.001	< 0.001
Low	REACTOME_DNA_REPLICATION_PRE_INITIATION	< 0.001	0.001	< 0.001	< 0.001	< 0.001	0.007	< 0.001	< 0.001	< 0.001	< 0.001	< 0.001	0.008	< 0.001	0.003
Low	REACTOME_REGULATION_OF_MITOTIC_CELL_CYCLE	< 0.001	< 0.001	< 0.001	< 0.001	< 0.001	< 0.001	< 0.001	< 0.001	< 0.001	< 0.001	< 0.001	0.003	< 0.001	< 0.001
Low	REACTOME_G1_S_DNA_DAMAGE_CHECKPOINTS	< 0.001	0.014	< 0.001	< 0.001	< 0.001	0.004	< 0.001	< 0.001	< 0.001	< 0.001	< 0.001	0.003	< 0.001	0.001
Low	REACTOME_DNA_REPLICATION	< 0.001	< 0.001	< 0.001	< 0.001	< 0.001	0.004	< 0.001	< 0.001	< 0.001	< 0.001	< 0.001	0.032	< 0.001	0.001
Low	REACTOME_DEGRADATION_OF_GLI1_BY_THE_PROTEASOME	< 0.001	0.007	< 0.001	0.001	< 0.001	0.004	< 0.001	< 0.001	< 0.001	< 0.001	< 0.001	< 0.001	< 0.001	0.001
Low	REACTOME_DEGRADATION_OF_BETA_CATENIN_BY_THE_DESTRUCTION_COMPLEX	< 0.001	0.014	< 0.001	0.003	< 0.001	0.008	< 0.001	< 0.001	0.003	< 0.001	< 0.001	0.002	< 0.001	0.016
Low	REACTOME_NEGATIVE_REGULATION_OF_NOTCH4_SIGNALING	< 0.001	0.001	< 0.001	< 0.001	< 0.001	< 0.001	< 0.001	< 0.001	< 0.001	< 0.001	< 0.001	< 0.001	< 0.001	< 0.001
Low	REACTOME_DEGRADATION_OF_AXIN	< 0.001	0.004	< 0.001	0.001	< 0.001	0.007	< 0.001	< 0.001	< 0.001	< 0.001	< 0.001	0.001	< 0.001	0.003
Low	REACTOME_ANTIGEN_PROCESSING_CROSS_PRESENTATION	< 0.001	< 0.001	< 0.001	0.002	< 0.001	0.004	< 0.001	< 0.001	< 0.001	< 0.001	< 0.001	0.002	< 0.001	< 0.001
Low	REACTOME_CROSS_PRESENTATION_OF_SOLUBLE_EXOGENOUS_ANTIGENS_ENDOSOMES	< 0.001	0.002	< 0.001	0.001	< 0.001	< 0.001	< 0.001	< 0.001	< 0.001	< 0.001	< 0.001	< 0.001	< 0.001	< 0.001
Low	REACTOME_ACTIVATION_OF_NF_KAPPAB_IN_B_CELLS	< 0.001	0.036	< 0.001	0.001	< 0.001	0.032	< 0.001	< 0.001	< 0.001	< 0.001	< 0.001	0.002	< 0.001	0.006
Low	REACTOME_TNFR2_NON_CANONICAL_NF_KB_PATHWAY	< 0.001	0.002	< 0.001	0.001	< 0.001	0.026	< 0.001	< 0.001	< 0.001	< 0.001	< 0.001	0.003	< 0.001	0.001
Low	KEGG_OXIDATIVE_PHOSPHORYLATION	< 0.001	< 0.001	< 0.001	< 0.001	< 0.001	< 0.001	< 0.001	< 0.001	< 0.001	< 0.001	< 0.001	< 0.001	< 0.001	< 0.001
Low	KEGG_PROTEASOME	< 0.001	0.001	< 0.001	< 0.001	< 0.001	< 0.001	< 0.001	< 0.001	< 0.001	< 0.001	< 0.001	< 0.001	< 0.001	< 0.001
Low	REACTOME_MITOCHONDRIAL_FATTY_ACID_BETA_OXIDATION	< 0.001	0.001	< 0.001	< 0.001	0.001	0.004	< 0.001	< 0.001	< 0.001	< 0.001	< 0.001	< 0.001	< 0.001	0.003
Low	REACTOME_RESPIRATORY_ELECTRON_TRANSPORT	< 0.001	< 0.001	< 0.001	< 0.001	< 0.001	< 0.001	< 0.001	< 0.001	< 0.001	< 0.001	< 0.001	< 0.001	< 0.001	< 0.001
Low	REACTOME_ASSEMBLY_OF_THE_PRE_REPLICATIVE_COMPLEX	< 0.001	0.001	< 0.001	< 0.001	< 0.001	0.005	< 0.001	< 0.001	< 0.001	< 0.001	< 0.001	0.012	< 0.001	0.002
Low	REACTOME_SCF_SKP2_MEDIATED_DEGRADATION_OF_P27_P21	< 0.001	0.007	< 0.001	< 0.001	< 0.001	< 0.001	< 0.001	< 0.001	< 0.001	< 0.001	< 0.001	< 0.001	< 0.001	< 0.001
Low	REACTOME_ACTIVATION_OF_APC_C_AND_APC_C:CDC20_MEDIATED_DEGRADATION_OF_MITOTIC_PROTEINS	< 0.001	< 0.001	< 0.001	< 0.001	< 0.001	< 0.001	< 0.001	< 0.001	< 0.001	< 0.001	< 0.001	0.001	< 0.001	< 0.001
Low	REACTOME_BASE_EXCISION_REPAIR_AP_SITE_FORMATION	< 0.001	0.008	< 0.001	0.001	0.008	0.006	0.002	< 0.001	0.001	< 0.001	0.026	0.010	< 0.001	0.002
Low	REACTOME_THE_ROLE_OF_GTSE1_IN_G2_M_PROGRESSION_AFTER_G2_CHECKPOINT	< 0.001	0.002	< 0.001	< 0.001	< 0.001	< 0.001	< 0.001	< 0.001	< 0.001	< 0.001	< 0.001	< 0.001	< 0.001	< 0.001
Low	REACTOME_ORC1_REMOVAL_FROM_CHROMATIN	< 0.001	0.003	< 0.001	< 0.001	< 0.001	0.004	< 0.001	< 0.001	< 0.001	< 0.001	< 0.001	0.010	< 0.001	0.003
Low	REACTOME_REGULATION_OF_APOPTOSIS	< 0.001	0.001	< 0.001	< 0.001	< 0.001	0.001	< 0.001	< 0.001	< 0.001	< 0.001	< 0.001	0.001	< 0.001	< 0.001

Low	REACTOME_BASE_EXCISION_REPAIR	< 0.001	< 0.001	< 0.001	< 0.001	0.003	0.004	< 0.001	< 0.001	< 0.001	< 0.001	< 0.001	0.012	< 0.001	< 0.001
Low	REACTOME_CDK_MEDIATED_PHOSPHORYLATION_AND_REMOVAL_OF_CDC6	< 0.001	< 0.001	< 0.001	< 0.001	< 0.001	< 0.001	< 0.001	< 0.001	< 0.001	< 0.001	< 0.001	< 0.001	< 0.001	< 0.001
Low	REACTOME_CYCLIN_A:CDK2_ASSOCIATED_EVENTS_AT_S_PHASE_ENTRY	< 0.001	0.041	< 0.001	0.001	< 0.001	0.044	< 0.001	< 0.001	< 0.001	< 0.001	0.010	0.007	< 0.001	0.013
Low	REACTOME_FBXL7_DOWN_REGULATES_AURKA_DURING_MITOTIC_ENTRY_AND_IN_EARLY_MITOSIS	< 0.001	0.001	< 0.001	< 0.001	< 0.001	< 0.001	< 0.001	< 0.001	< 0.001	< 0.001	< 0.001	< 0.001	< 0.001	< 0.001
Low	REACTOME_STABILIZATION_OF_P53	< 0.001	0.001	< 0.001	< 0.001	< 0.001	0.002	< 0.001	< 0.001	< 0.001	< 0.001	< 0.001	0.001	< 0.001	< 0.001
Low	REACTOME_SWITCHING_OF_ORIGINS_TO_A_POST_REPLICATIVE_STATE	< 0.001	< 0.001	< 0.001	< 0.001	< 0.001	0.001	< 0.001	< 0.001	< 0.001	< 0.001	< 0.001	0.012	< 0.001	< 0.001
Low	REACTOME_PCP_CE_PATHWAY	< 0.001	0.033	< 0.001	0.011	< 0.001	0.007	< 0.001	< 0.001	0.005	< 0.001	0.003	0.002	< 0.001	0.007
Low	REACTOME_HEDGEHOG_LIGAND_BIOGENESIS	< 0.001	0.001	< 0.001	0.001	< 0.001	0.004	< 0.001	< 0.001	< 0.001	< 0.001	< 0.001	< 0.001	< 0.001	< 0.001
Low	REACTOME_DEGRADATION_OF_DVL	< 0.001	0.001	< 0.001	< 0.001	< 0.001	0.001	< 0.001	< 0.001	< 0.001	< 0.001	< 0.001	< 0.001	< 0.001	< 0.001
Low	REACTOME_ASYMMETRIC_LOCALIZATION_OF_PCP_PROTEINS	< 0.001	0.009	< 0.001	< 0.001	< 0.001	0.004	< 0.001	< 0.001	0.001	< 0.001	< 0.001	0.001	< 0.001	0.007
Low	REACTOME_RUNX1_REGULATES_TRANSCRIPTION_OF_GENES_INVOLVED_IN_DIFFERENTIATION_OF_HSCS	< 0.001	0.005	< 0.001	< 0.001	< 0.001	0.004	< 0.001	< 0.001	< 0.001	< 0.001	0.001	0.001	< 0.001	< 0.001
Low	REACTOME_REGULATION_OF_RUNX3_EXPRESSION_AND_ACTIVITY	< 0.001	0.001	< 0.001	0.001	< 0.001	0.002	< 0.001	< 0.001	< 0.001	< 0.001	< 0.001	< 0.001	< 0.001	0.003
Low	REACTOME_VIF_MEDIATED_DEGRADATION_OF_APOBEC3G	< 0.001	< 0.001	< 0.001	< 0.001	< 0.001	< 0.001	< 0.001	< 0.001	< 0.001	< 0.001	< 0.001	< 0.001	< 0.001	< 0.001
Low	REACTOME_DECTIN_1_MEDIATED_NONCANONICAL_NF_KB_SIGNALING	< 0.001	< 0.001	< 0.001	< 0.001	< 0.001	0.004	< 0.001	< 0.001	< 0.001	< 0.001	< 0.001	< 0.001	< 0.001	< 0.001
Low	REACTOME_REGULATION_OF_RUNX2_EXPRESSION_AND_ACTIVITY	< 0.001	0.026	< 0.001	0.003	< 0.001	0.006	< 0.001	< 0.001	< 0.001	< 0.001	0.001	0.009	< 0.001	0.002
Low	REACTOME_REGULATION_OF_MRNA_STABILITY_BY_PROTEINS_THAT_BIND_AU_RICH_ELEMENTS	< 0.001	0.001	< 0.001	< 0.001	< 0.001	0.006	< 0.001	< 0.001	0.001	< 0.001	< 0.001	0.014	< 0.001	0.001
Low	REACTOME_METABOLISM_OF_POLYAMINES	< 0.001	0.003	< 0.001	< 0.001	< 0.001	< 0.001	< 0.001	< 0.001	< 0.001	< 0.001	< 0.001	< 0.001	< 0.001	< 0.001
Low	REACTOME_AUF1_HNRNP_D0_BINDS_AND_DESTABILIZES_MRNA	< 0.001	0.001	< 0.001	< 0.001	< 0.001	0.001	< 0.001	< 0.001	< 0.001	< 0.001	< 0.001	< 0.001	< 0.001	< 0.001
Low	REACTOME_CELLULAR_RESPONSE_TO_HYPOXIA	< 0.001	0.017	< 0.001	0.002	< 0.001	0.036	< 0.001	< 0.001	< 0.001	< 0.001	< 0.001	0.003	< 0.001	0.016
Low	REACTOME_MITOCHONDRIAL_TRANSLATION	< 0.001	< 0.001	< 0.001	< 0.001	< 0.001	< 0.001	< 0.001	< 0.001	< 0.001	< 0.001	< 0.001	< 0.001	< 0.001	< 0.001
Low	REACTOME_THE_CITRIC_ACID_TCA_CYCLE_AND_RESPIRATORY_ELECTRON_TRANSPORT	< 0.001	< 0.001	< 0.001	< 0.001	< 0.001	< 0.001	< 0.001	< 0.001	< 0.001	< 0.001	< 0.001	< 0.001	< 0.001	< 0.001
Low	REACTOME_MITOCHONDRIAL_PROTEIN_IMPORT	< 0.001	< 0.001	< 0.001	< 0.001	< 0.001	< 0.001	< 0.001	< 0.001	< 0.001	< 0.001	< 0.001	0.001	< 0.001	< 0.001
Low	REACTOME_RESPIRATORY_ELECTRON_TRANSPORT_ATP_SYNTHESIS_BY_CHEMIOSMOTIC_COUPLING_AND_HEAT_PRODUCTION_BY_UNCOUPLING_PROTEINS	< 0.001	< 0.001	< 0.001	< 0.001	< 0.001	< 0.001	< 0.001	< 0.001	< 0.001	< 0.001	< 0.001	< 0.001	< 0.001	< 0.001
Low	REACTOME_UCH_PROTEINASES	< 0.001	0.012	< 0.001	0.010	< 0.001	0.017	< 0.001	< 0.001	< 0.001	< 0.001	< 0.001	0.003	< 0.001	0.007

Low	REACTOME_COMPLEX_I_BIOGENESIS	< 0.001	< 0.001	< 0.001	< 0.001	< 0.001	< 0.001	< 0.001	< 0.001	< 0.001	< 0.001	< 0.001	< 0.001	< 0.001	< 0.001
Low	REACTOME_PROTEIN_LOCALIZATION	< 0.001	< 0.001	< 0.001	< 0.001	< 0.001	0.001	< 0.001	< 0.001	< 0.001	< 0.001	< 0.001	0.002	< 0.001	< 0.001
Low	REACTOME_ABC_TRANSPORTER_DISORDERS	< 0.001	0.001	< 0.001	0.001	< 0.001	0.007	< 0.001	< 0.001	< 0.001	< 0.001	< 0.001	0.001	< 0.001	0.003
Low	REACTOME_RESOLUTION_OF_ABASIC_SITES_AP_SITES	< 0.001	< 0.001	< 0.001	< 0.001	0.004	0.014	< 0.001	< 0.001	< 0.001	< 0.001	< 0.001	0.037	< 0.001	0.008
Low	REACTOME_DEFECTIVE_CFTR_CAUSES_CYSTIC_FIBROSIS	< 0.001	0.001	< 0.001	< 0.001	< 0.001	0.004	< 0.001	< 0.001	< 0.001	< 0.001	< 0.001	< 0.001	< 0.001	0.001
Low	KEGG_PARKINSONS_DISEASE	< 0.001	< 0.001	< 0.001	< 0.001	< 0.001	< 0.001	< 0.001	< 0.001	< 0.001	< 0.001	< 0.001	< 0.001	< 0.001	< 0.001
Low	KEGG_ALZHEIMERS_DISEASE	< 0.001	< 0.001	< 0.001	< 0.001	< 0.001	< 0.001	< 0.001	< 0.001	< 0.001	< 0.001	< 0.001	0.001	< 0.001	< 0.001
Low	KEGG_HUNTINGTONS_DISEASE	< 0.001	< 0.001	< 0.001	< 0.001	< 0.001	< 0.001	< 0.001	< 0.001	< 0.001	< 0.001	< 0.001	0.003	< 0.001	< 0.001
High	REACTOME_SIGNALING_BY_VEGF	0.012	0.005	0.002	< 0.001	0.005	0.001	0.003	0.001	0.001	0.002	0.045	1.000	0.002	0.022
High	KEGG_ADHERENS_JUNCTION	< 0.001	0.004	0.001	< 0.001	< 0.001	< 0.001	0.012	< 0.001	< 0.001	< 0.001	0.003	1.000	0.011	< 0.001
High	REACTOME_ECM_PROTEOGLYCANS	< 0.001	0.004	< 0.001	< 0.001	0.001	0.001	< 0.001	< 0.001	< 0.001	< 0.001	1.000	1.000	< 0.001	< 0.001
High	REACTOME_EXTRACELLULAR_MATRIX_ORGANIZATION	< 0.001	0.005	< 0.001	< 0.001	0.012	0.013	< 0.001	< 0.001	< 0.001	< 0.001	1.000	1.000	< 0.001	0.009
High	KEGG_HEDGEHOG_SIGNALING_PATHWAY	0.005	0.018	0.026	0.003	1.000	0.022	0.007	< 0.001	< 0.001	< 0.001	0.008	1.000	< 0.001	0.010
High	REACTOME_NOTCH4_INTRACELLULAR_DOMAIN_REGULATES_TRANSCRIPTION	0.002	0.008	0.013	0.017	0.001	0.015	< 0.001	< 0.001	0.001	0.001	1.000	1.000	0.007	0.004
High	KEGG_WNT_SIGNALING_PATHWAY	0.021	0.005	1.000	0.001	0.026	0.026	0.004	0.002	0.001	< 0.001	0.036	1.000	0.003	0.002
High	KEGG_TGF_BETA_SIGNALING_PATHWAY	< 0.001	0.004	1.000	0.001	0.002	0.002	0.003	0.002	0.015	0.002	0.041	1.000	0.003	< 0.001
High	REACTOME_MET_PROMOTES_CELL_MOTILITY	< 0.001	< 0.001	0.001	< 0.001	0.001	< 0.001	0.001	< 0.001	< 0.001	< 0.001	0.043	1.000	< 0.001	< 0.001
High	REACTOME_SIGNALING_BY_NTRK1_TRKA	0.008	0.010	0.022	< 0.001	0.007	0.023	0.021	0.019	0.001	0.009	1.000	1.000	0.002	0.014
High	REACTOME_SIGNALING_BY_MET	0.001	0.003	0.027	< 0.001	0.018	0.010	0.012	< 0.001	< 0.001	0.002	1.000	1.000	0.003	0.007
High	REACTOME_MET_ACTIVATES_PTK2_SIGNALING	< 0.001	0.006	0.001	< 0.001	0.007	0.001	< 0.001	< 0.001	< 0.001	< 0.001	1.000	1.000	< 0.001	< 0.001
High	KEGG_RENAL_CELL_CARCINOMA	0.005	0.006	0.013	< 0.001	0.001	0.006	0.023	0.020	< 0.001	0.031	0.049	1.000	0.001	0.025
High	KEGG_SMALL_CELL_LUNG_CANCER	0.007	0.002	0.015	0.007	0.006	0.001	0.001	0.006	0.001	0.021	1.000	1.000	0.011	0.006
High	KEGG_PATHWAYS_IN_CANCER	0.001	0.002	0.022	0.001	0.006	0.003	0.002	< 0.001	< 0.001	< 0.001	1.000	1.000	< 0.001	0.002
High	KEGG_FOCAL_ADHESION	< 0.001	0.004	< 0.001	< 0.001	0.001	< 0.001	< 0.001	< 0.001	< 0.001	< 0.001	0.041	1.000	< 0.001	< 0.001
High	REACTOME_SIGNALING_BY_PDGF	< 0.001	0.003	< 0.001	< 0.001	0.001	0.001	< 0.001	< 0.001	< 0.001	< 0.001	1.000	1.000	< 0.001	< 0.001
High	REACTOME_NON_INTEGRIN_MEMBRANE_ECM_INTERACTIONS	< 0.001	0.004	0.001	< 0.001	0.001	< 0.001	0.001	< 0.001	< 0.001	< 0.001	0.022	1.000	< 0.001	< 0.001
High	REACTOME_COLLAGEN_BIOSYNTHESIS_AND_MODIFYING_ENZYMES	< 0.001	0.012	< 0.001	< 0.001	0.034	0.032	0.004	< 0.001	< 0.001	< 0.001	1.000	1.000	< 0.001	0.022
High	REACTOME_INTEGRIN_CELL_SURFACE_INTERACTIONS	< 0.001	0.018	0.017	< 0.001	0.008	0.004	< 0.001	< 0.001	< 0.001	< 0.001	1.000	1.000	< 0.001	0.014
High	REACTOME_LAMININ_INTERACTIONS	< 0.001	0.009	< 0.001	< 0.001	< 0.001	< 0.001	< 0.001	< 0.001	< 0.001	< 0.001	1.000	1.000	< 0.001	< 0.001
High	REACTOME_O_GLYCOSYLATION_OF_TSR_DOMAIN_CONTAINING_PROTEINS	< 0.001	0.003	0.015	< 0.001	0.007	0.013	< 0.001	< 0.001	< 0.001	< 0.001	1.000	1.000	< 0.001	0.031

High	REACTOME_COLLAGEN_CHAIN_TRIMERIZATION	< 0.001	0.018	0.001	< 0.001	0.013	0.006	< 0.001	< 0.001	< 0.001	< 0.001	1.000	1.000	< 0.001	0.005
High	REACTOME_ELASTIC_FIBRE_FORMATION	< 0.001	0.008	0.033	< 0.001	0.035	0.008	0.002	< 0.001	< 0.001	< 0.001	1.000	1.000	< 0.001	0.010
High	KEGG_ECM_RECEPTOR_INTERACTION	< 0.001	0.002	< 0.001	< 0.001	0.001	< 0.001	< 0.001	< 0.001	< 0.001	< 0.001	1.000	1.000	< 0.001	0.001
High	REACTOME_NCAM_SIGNALING_FOR_NEURITE_OUT_GROWTH	< 0.001	0.027	< 0.001	< 0.001	0.021	0.013	0.001	< 0.001	< 0.001	< 0.001	1.000	1.000	< 0.001	< 0.001
High	REACTOME_NEPHRIN_FAMILY_INTERACTIONS	0.010	0.045	0.022	0.003	0.007	0.007	0.023	< 0.001	0.009	0.037	1.000	1.000	0.008	0.014
High	REACTOME_BMAL1:CLOCK_NPAS2_ACTIVATES_CIRCADIAN_GENE_EXPRESSION	0.010	0.005	0.013	0.002	0.015	0.008	0.007	0.033	0.001	0.001	0.011	1.000	NA	0.041
High	REACTOME_NETRIN_1_SIGNALING	0.003	0.009	0.006	0.001	0.007	< 0.001	< 0.001	0.001	0.001	0.003	0.001	1.000	0.001	0.001
High	REACTOME_INTERLEUKIN_6_FAMILY_SIGNALING	0.006	0.018	0.043	< 0.001	0.007	0.012	0.044	0.007	0.002	0.006	0.029	1.000	0.004	0.006
High	KEGG_AXON_GUIDANCE	< 0.001	0.003	0.001	< 0.001	0.001	0.013	< 0.001	< 0.001	< 0.001	< 0.001	0.011	1.000	< 0.001	0.001
High	REACTOME_G_PROTEIN_MEDIATED_EVENTS	0.001	0.037	0.002	0.002	0.008	0.016	0.003	0.002	0.001	0.007	0.032	1.000	< 0.001	0.014
High	REACTOME_DOWNSTREAM_SIGNAL_TRANSDUCTION	0.005	0.003	0.002	0.001	0.007	0.005	0.005	0.004	0.004	0.002	1.000	1.000	< 0.001	0.009
High	REACTOME_SIGNALING_BY_NTRKS	0.017	0.018	0.007	0.002	0.013	0.039	0.021	0.013	0.001	0.008	1.000	1.000	0.001	0.022
High	REACTOME_SIGNALING_BY_RECEPTOR_TYROSINE_KINASES	0.001	0.017	0.020	0.001	0.018	0.010	0.003	< 0.001	0.001	0.002	1.000	1.000	0.001	0.013
